# Semi‐Dwarfing Reduced Height Genes Hardly Influenced Gluten Protein Composition While Extreme Dwarfing Genes Decreased Glutenins in Wheat

**DOI:** 10.1002/fsn3.70649

**Published:** 2025-07-30

**Authors:** Sabrina Geisslitz, Matías Schierenbeck, Andreas Börner, Katharina Anne Scherf

**Affiliations:** ^1^ Department of Bioactive and Functional Food Chemistry, Institute of Applied Biosciences Karlsruhe Institute of Technology (KIT) Karlsruhe Germany; ^2^ Leibniz Institute for Food Systems Biology at the Technical University of Munich Freising Germany; ^3^ Physiology and Cell Biology Department Leibniz Institute of Plant Genetics and Crop Plant Research (IPK) Seeland/OT Gatersleben Germany; ^4^ Genebank Department Leibniz Institute of Plant Genetics and Crop Plant Research (IPK) Seeland/OT Gatersleben Germany; ^5^ Professorship of Food Biopolymer Systems, TUM School of Life Sciences Technical University of Munich Freising Germany

**Keywords:** gliadin‐to‐glutenin‐ratio, Green Revolution, kernel morphology, near isogenic lines, Osborne fractionation, *Rht* genes

## Abstract

The introduction of reduced height (*Rht*) genes into wheat during the Green Revolution led to lower plant height, but their effect on protein composition was unknown. Therefore, the protein composition of near isogenic lines (NILs) of four *Triticum aestivum* genotypes with five different *Rht* allele/allele combinations was compared to the tall wild‐type (*rht*) by modified Osborne fractionation. The semi‐dwarfing (*Rht1*, *Rht2*) and the dwarf *Rht* gene combination (*Rht1 + 2*) only had a small effect on protein composition. The extreme dwarfing genes (*Rht3* and *Rht2 + 3*) decreased glutenin content leading to higher gliadin‐to‐glutenin ratios compared to the tall wild‐type (*rht*). A strong environmental influence on the protein composition was observed. The introduction of the semi‐dwarfing and dwarfing *Rht* genes (*Rht1*, *Rht2*, *Rht1 + 2*) that are predominantly present in modern wheat does not represent the primary factor contributing to the observed variation in protein composition between modern and pre‐Green Revolution wheat cultivars. The extreme dwarfs *Rht3* and *Rht2 + 3* are not recommended to be included in wheat breeding programs due to their lower glutenin content. The high number of harvest years and biological replicates strengthen our findings. To our best knowledge, this is the first study that conducted Osborne fractionation on NILs with different *Rht* alleles.

## Graphical abstract

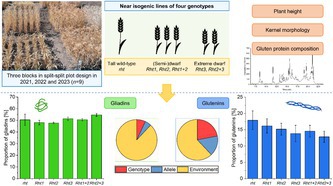

## 1 Introduction

One of the most important elements of the Green Revolution was the introduction of *reduced height* (*Rht*) genes in wheat ( *Triticum aestivum* L.) resulting in a higher harvest index, less tendency to lodging due to lower plant height, and increased fertilizer use efficiency (Wuerschum et al. 2017). Nowadays, more than 70% of wheat that is grown worldwide carries at least one of the semi‐dwarfing genes *Rht‐B1* (*Rht1*) or *Rht‐D1* (*Rht2*) (Evans 1998; Wuerschum et al. 2017).

In the last 50 years, breeding not only aimed to improve agronomic performance, but also end‐use quality and nutritional value of cereals. Wheat is mostly consumed as bread. Among others, the gluten proteins play a crucial role for baking quality as the flour forms an elastic and viscous dough when mixed with water (Sharma et al. 2020; Wieser et al. 2023). Gluten proteins are divided into monomeric gliadins and polymeric glutenins, and the gluten network is formed due to their interplay. High levels of glutenins, particularly high‐molecular‐weight glutenin subunits (HMW‐GS), are associated with stronger dough due to their contribution to dough elasticity and strength. In contrast, low‐molecular‐weight glutenin subunits (LMW‐GS) also contribute to dough properties but to a lesser extent. Gliadins enhance dough extensibility and viscosity (Wieser et al. 2023). As a result, the ratio between gliadins and glutenins defines the behavior of dough and the baking quality. Currently, the knowledge on the exact correlation between gluten protein composition and baking quality (e.g., specific bread volume, height‐to‐width‐ratio of the bread) remains uncertain. Some studies have indicated a strong correlation (Thanhaeuser et al. 2014; Wieser and Kieffer 2001) and others reported no correlation (Jahn et al. 2024; Schuster et al. 2023). Nevertheless, it is for sure that a large excess of gliadins over glutenins leads to very soft doughs and a low bread volume. Typical gliadin‐to‐glutenin ratios are 1.5–3.2 for wheat (Schuster et al. 2023; Thanhaeuser et al. 2014).

However, gluten is not only associated with baking quality, but gluten proteins also trigger wheat‐related disorders, such as celiac disease, wheat allergy, and wheat‐dependent exercise‐induced anaphylaxis (WDEIA) (reviewed by Wieser et al. (2020) and Kumar et al. (2024)). Certain gliadin types (e.g., α‐gliadins containing the 33‐mer peptide) and ω5‐gliadins are implicated in celiac disease and WDEIA, respectively. Due to the increasing prevalence of wheat‐related disorders during the last at least 40 years, one hypothesis is that Green Revolution breeding progress changed gluten protein composition, leading to a higher immunoreactive potential.

Overall, breeding indeed changed the gluten protein composition. Cultivars that were commonly grown before the Green Revolution had a lower glutenin content and a higher gliadin‐to‐glutenin ratio compared to cultivars commonly used after the Green Revolution (Call, Kapeller, et al. 2020; Pronin et al. 2020). Furthermore, landraces that were subject to modern breeding only to a very low extent had lower glutenin content and higher gliadin‐to‐glutenin ratios compared to modern cultivars (Jahn et al. 2024). A closer look at peptides that contain celiac disease active epitopes showed that cultivars used before and after the Green Revolution had almost the same content (Pronin et al. 2021).

Breeding is a complex procedure, and not only did the introduction of *Rht* genes during the Green Revolution take place. In addition, *Rht* dwarfing alleles play an essential role in hybrid wheat seed production due to their negative effects on floral traits such as anther extrusion, anther length, and anther filament length that are related to cross‐pollination efficiency (Boeven et al. 2016; Schierenbeck et al. 2024). To analyze the effect of specific genetic changes (here: *Rht* genes) on defined properties (e.g., plant height, grain yield, protein composition), near isogenic lines (NILs) can be used because they differ almost only in the target genes and are otherwise genetic homologues. Thus, NILs of four different cultivars (April Bearded, Bersee, Maris Huntsman and Maris Widgeon) with five different *Rht* alleles/combinations and the respective tall wild‐type have been analyzed already for different properties (e.g., plant height, agronomic and kernel morphology, floral traits, response to osmotic stress) (Börner et al. 1993; Flintham et al. 1997; Landjeva et al. 2008; Schierenbeck et al. 2024). The *Rht* alleles/combinations covered the tall wild‐type (*rht*, *Rht‐B1a/Rht‐D1a*), the semi‐dwarfs *Rht1* (*Rht‐B1b/Rht‐D1a*) and *Rht2* (*Rht‐B1a/Rht‐D1b*), the dwarf *Rht1 + 2* (*Rht‐B1b/RhtD1b*), and the extreme dwarfs *Rht3* (*Rht‐B1c/Rht‐D1a*) and *Rht2 + 3* (*Rht‐B1c/Rht‐D1b*) (Börner et al. 1993; Schierenbeck et al. 2024). The *Rht* alleles reduced plant height by up to 64%, decreased thousand kernel weight by up to 32% due to smaller kernels, but showed a differential impact on the kernel number per square meter depending on the degree of dwarfing imposed by each *Rht* gene and the environmental conditions explored (Börner et al. 1993; Schierenbeck et al. 2024). Compared to the wild‐type, increases in grain number of 20%–26% for *Rht1* and *Rht2*, 15% for *Rht1 + 2*, and 7% for *Rht3* were observed. On the other hand, reductions of 25% in grain number per area were reported for the extreme dwarf *Rht2 + 3* due to a lower above‐ground biomass accumulation and reduced leaf area index (Schierenbeck et al. 2024). However, it is currently not known if and how *Rht* genes changed protein composition, especially gluten protein composition. Only one study characterized gluten proteins from NILs that contained semi‐dwarf *Rht1*, *Rht2*, and *Rht8* (Jobson et al. 2020). Here, glutenins and especially HMW‐GS were more abundant in the semi‐dwarf lines compared to the tall wild‐type. However, both the absolute content and the ratio of gluten proteins are important for assessing wheat quality and potential health impacts.

The aim of our study was to elucidate the effect of different *Rht* genes in four genotypes on the gluten protein composition. It should be elucidated if the introduction of *Rht* genes during the Green Revolution increased glutenin content and decreased the gliadin‐to‐glutenin ratio, as this is true for cultivars that are used after the Green Revolution compared to cultivars before. Second, there was the assumption that breeding changed gluten protein composition and increased, as a consequence, the immunoreactive potential of wheat. To cover the effect of the environment, three harvest years with three biological replicates each were included.

## 2 Materials and Methods

### 2.1 Plant Material

Near isogenic lines (NILs) carrying the alleles/combinations *Rht1*, *Rht2*, *Rht3*, *Rht1 + 2*, *Rht2 + 3*, and *rht* (tall wild‐type) in the genetic backgrounds of the winter wheat cultivars April Bearded, Bersee, Maris Huntsman, and Maris Widgeon were selected (Börner et al. 1993; Schierenbeck et al. 2024). The lines were developed by recurrent backcrossing as described in Youssefian et al. (1992). Field trials were performed over three consecutive growing seasons (2020/2021, 2021/2022 and 2022/2023) at the Leibniz Institute of Plant Genetics and Crop Plant Research (IPK) in Gatersleben, Germany (11°16′ LE; 51°49′ LN). A randomized complete block design was employed, with a split‐split‐plot arrangement of treatments with three blocks in each year (three biological replicates per year). Each plot was 1.2 m^2^ (1 m long by 1.2 m wide), comprising six rows. Standard agronomic practices for managing insects, fungal diseases, and weeds were implemented throughout the crop cycle. The experimental design is already reported in detail in Schierenbeck et al. (2024). In short, alleles (subplots) and genotypes (sub‐subplots) were considered as fixed effects, and the environments and biological replicates (main plots) as random effects.

In early spring (February/March) of each growing season, a soil nutrient analysis of the experimental fields of IPK was done by AGROLAB Agrarzentrum GmbH (Leinefelde‐Worbis, Germany). The average values of phosphorus (P), potassium (K), magnesium (Mg), nitrate nitrogen (NO_3_‐N) and ammonia nitrogen (NH_4_‐N) of soil are given in Table S1.

Kernels were milled to wholemeal flours with a tube mill (IKA, Staufen, Germany). About 20 g kernels were milled three times for 1 min with a break of 1 min in between. The flour was stored for at least two weeks before analysis, but not longer than six weeks.

### 2.2 Plant Height, Grain Yield, and Kernel Morphology

Plant height was measured from the ground to the tip of the spikes (excluding awns) at the early dough stage (Schierenbeck et al. 2024). After harvesting, 200 randomly selected kernels from each plot (per biological replicate and year, Table S2) were employed to assess kernel morphological traits including kernel length, kernel width, kernel surface area, and thousand kernel weight, utilizing the MARVIN Digital Seed Analyzer (MARViTECH GmbH, Germany) (Schierenbeck et al. 2021). Grain number per square meter (GN) was calculated by counting the spikes in 2 m central rows and threshing and counting the grains in 20 spikes per plot. Grain yield per plot (GY) in g/m^2^ was calculated as the product of GN and individual kernel weight. Data of plant height, GN, and thousand kernel weight from samples harvested in 2021 and 2022 are already reported in Schierenbeck et al. (2024).

### 2.3 Crude Protein Content by Dumas

The crude protein content of the flours (nitrogen × 5.71) was determined by the Dumas method using a Dumatherm Nitrogen analyzer (Gerhardt Instruments, Königswinter, Germany) following ICC Standard No. 167. All determinations were done in triplicates to obtain three technical replicates each (Table S2).

### 2.4 Extraction of Osborne Fractions

The Osborne fractions albumins/globulins, gliadins, and glutenins were extracted according to the modified Osborne fractionation as reported by Wieser et al. (1998). Albumins/globulins were extracted twice with 0.4 mol/L NaCl/0.067 mol/L Na_2_HPO_4_/KH_2_PO_4_ (pH 7.6, 1 mL), gliadins three times with 60% (v/v) ethanol (0.5 mL) and glutenins twice with 50% (v/v) 1‐propanol/2 mol/L urea/0.05 mol/L TRIS–HCl (pH 7.5)/1% (w/v) dithiothreitol under nitrogen (1 mL). Each extraction step was carried out with vortexing (2 min) and stirring (albumins/globulins and gliadins 10 min at 22°C and glutenins 30 min at 60°C). After centrifugation for 25 min at 3550 rcf and 22°C, the supernatants were collected in 2 mL flasks, and the flasks were filled with the respective extraction solution. The solutions were filtered through a 0.45 μm syringe filter with a regenerated cellulose membrane (WICOM, Heppenheim, Germany) directly into vials. Two separate extraction experiments were carried out for each flour sample to obtain two technical replicates each (Table S2).

### 2.5 Reversed‐Phase High‐Performance Liquid Chromatography

The albumin/globulin, gliadin, and glutenin extracts were separated using a SIL‐40C HPLC (Shimadzu, Nakagyo‐ku, Kyoto, Japan) and the following parameters: Stationary phase: YMC Triart Bio C_18_, 150 mm × 2.1 mm, 3 μm (YMC Europe, Dinslaken, Germany); mobile phase A: 0.1% trifluoroacetic acid in water and B: 0.1% trifluoroacetic acid in acetonitrile; flow rate: 0.4 mL/min; column temperature: 60°C; injection volume: 10 μL for albumins/globulins, 10 μL for gliadins, and 20 μL for glutenins; gradient for albumins/globulins: 0–0.4 min, 0% B; 0.5 min, 20% B; 8 min, 60% B; 8.1 min, 90% B; 8.1–13 min, 90% B; 13.1–27 min, 0% B and gradient for gliadins and glutenins: 0–0.4 min, 5% B; 0.5 min, 30% B; 16 min, 60% B; 16.1–22.1 min, 90% B; 22.2 min, 5% B; 22.2–30 min, 5% B; detection: UV absorbance at 210 nm. The proteins were quantitated with an external calibration using Prolamin Working Group (PWG)‐gliadin (distributed by Arbeitsgemeinschaft Getreideforschung e.V., Detmold, Germany) at a concentration of 2.5 mg/mL, as described earlier (van Eckert et al. 2006). Chromatograms of gliadins were divided into ω5‐, ω1,2‐, α‐ and γ‐gliadins and those of glutenins into ωb‐gliadins, LMW‐GS, and HMW‐GS according to Schalk, Lexhaller, et al. (2017).

### 2.6 Statistics

Statistical analysis was conducted with OriginPro 2022 (OriginLab Corporation Northampton, MA, USA). Differences of *Rht1*, *Rht2*, *Rht3*, *Rht1 + 2*, and *Rht2 + 3* to *rht*, respectively, were tested by two‐sample *t*‐test (*n* = 18), whereas equal variance was not assumed (Welch correction). Linear fits were performed for proportions of gliadin types and glutenin subunits (*n* = 6). Nonlinear curve fit was used with the model parabola and Levenberg Marquardt as the iteration algorithm (*n* = 24). Pearson correlation coefficients (*r*) were calculated using not averaged values (*n* = 216). Three‐way ANOVA with the factors genotype (April Bearded, Bersee, Maris Huntsman and Maris Widgeon), allele (*rht*, *Rht1*, *Rht2*, *Rht3*, *Rht1 + 2*, *Rht2 + 3*) and environment (2021, 2022, 2023) was conducted at a significance level of *p* ≤ 0.05 without considering repeated measurements.

## 3 Results and Discussion

Four sets of NILs (genotypes April Bearded, Bersee, Maris Huntsman and Maris Widgeon) were included in the study that were available as the tall wild‐type (*rht*) and five *Rht* alleles/combinations (semi‐dwarfs *Rht1* and *Rht2*, dwarf *Rht1 + 2*, and extreme dwarfs *Rht3* and *Rht2 + 3*). The sample sets harvested in 2021, 2022, and 2023 were analyzed for plant height, kernel morphology, as well as crude protein content and protein composition. If no year is explicitly stated, the mean averaged over three biological replicates and three years is presented (Table S2).

### 3.1 Plant Height

**FIGURE 1:**
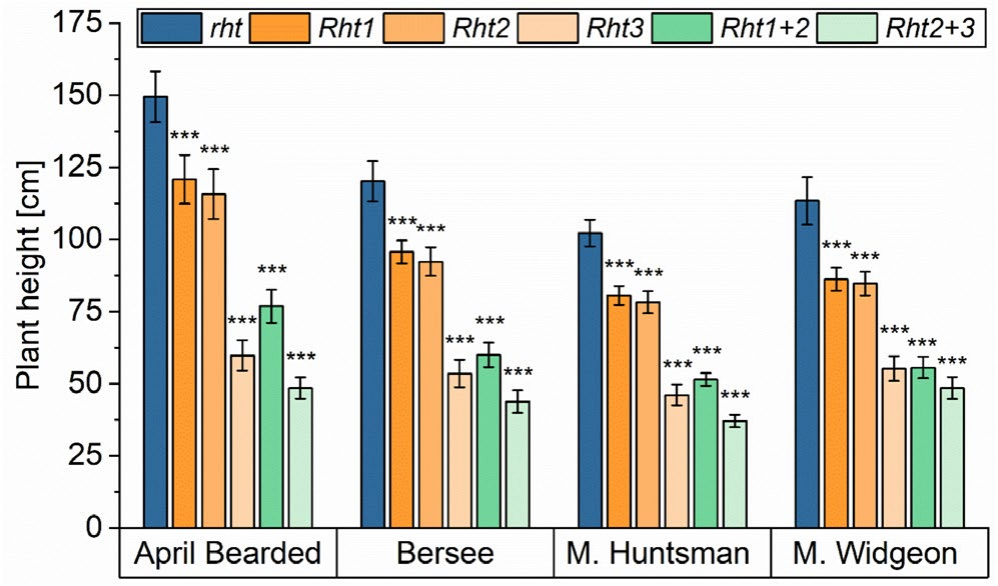
Plant height of NILs of the four genotypes April Bearded, Bersee, Maris (M.) Huntsman, and Maris (M.) Widgeon with different *Rht* alleles. *Rht1* (semi‐dwarf), *Rht2* (semi‐dwarf), *Rht3* (extreme dwarf), *Rht1 + 2* (dwarf), and *Rht2 + 3* (extreme dwarf) are compared to wild‐type *rht* (tall). Mean of three years and three biological replicates per year (2021, 2022, and 2023; *n* = 9). Significant differences to the control are marked with asterisks (*t*‐test, ***p < 0.001).

The wild‐type (*rht*) of all four sets of NILs had the highest plant height, and all *Rht* alleles significantly reduced plant height (Figure 1, Table S3). Exemplary photos of the field trials are shown in Figure S1. In agreement with previous studies (Börner et al. 1993; Gooding et al. 2012; Schierenbeck et al. 2024), the semi‐dwarf *Rht1* and *Rht2* decreased plant height between 19% and 25% compared to the respective wild‐type. A reduction of 49%–51% was observed for dwarf *Rht1 + 2* when compared to *rht*. The most extreme effect was documented for the extreme dwarf *Rht3* and *Rht2 + 3* because plant height was reduced by 50%–60% and 57%–68%, respectively, compared to the wild‐type. The reduction of plant height is due to the reduction of internode length as a result of an inhibition in the gibberellin signaling pathway (Du et al. 2018; Flintham et al. 1997).

### 3.2 Kernel Morphology

**FIGURE 2:**
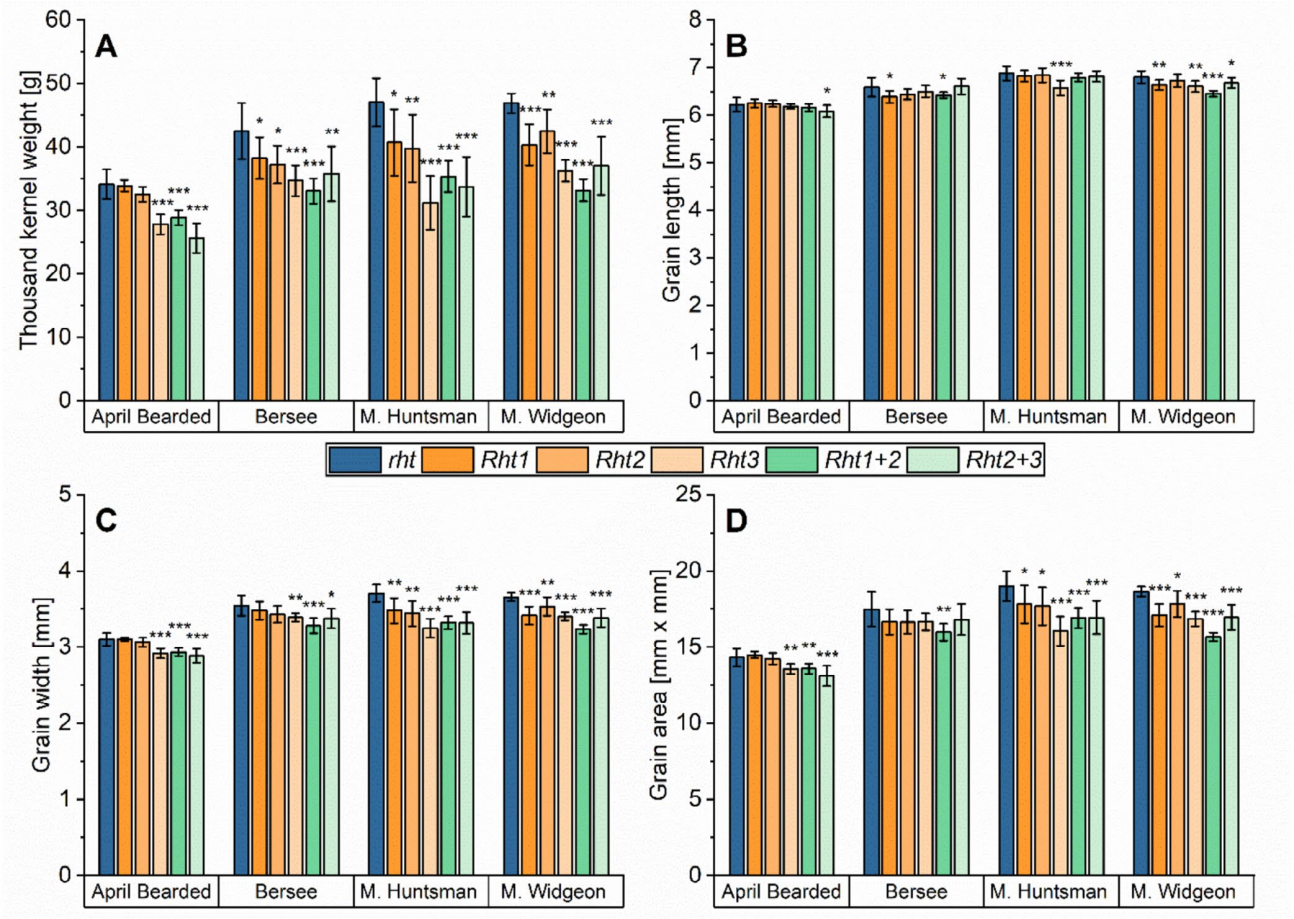
Kernel morphology of NILs of the four genotypes April Bearded, Bersee, Maris (M.) Huntsman and Maris (M.) Widgeon with different *Rht* alleles. *Rht1* (semi‐dwarf), *Rht2* (semi‐dwarf), *Rht3* (extreme dwarf), *Rht1 + 2* (dwarf), and *Rht2 + 3* (extreme dwarf) are compared to wild‐type *rht* (tall). (A) Thousand kernel weight; (B) grain length; (C) grain width; (D) grain area. Mean of three years and three biological replicates per year (2021, 2022, and 2023; *n* = 9). Significant differences to the control are marked with asterisks (*t*‐test, *p ≤ 0.05; **p < 0.01; ***p < 0.001).

Thousand kernel weight was highest for the wild‐type (*rht*) within each set of NILs compared to the respective *Rht* alleles (Figure 2A, Table S3). Again, the semi‐dwarf *Rht1* (1%–14%) and *Rht2* (5%–15%) decreased thousand kernel weight to a lower extent than the dwarf *Rht1 + 2* (15%–29%) and the extreme dwarfs *Rht3* (18%–34%) and *Rht2 + 3* (16%–28%). With the exception of *Rht1* and *Rht2* of April Bearded, the decrease was significant. Grain length (Figure 2B) was less affected (1%–5%) by the different *Rht* alleles compared to grain width (Figure 2C). Exemplary kernel photos of Maris Huntsman—the genotype with the most pronounced changes—are shown in Figure S2. Overall, only some significant differences were observed for grain length without clear trends. In contrast, the dwarf *Rht1 + 2* (5%–12%) and extreme dwarf *Rht3* (4%–12%) and *Rht2 + 3* (5%–10%) reduced grain width significantly in all four genotypes. This consequently led to lower grain areas (Figure 2D), which were reduced by the *Rht* alleles with the exception of *Rht1* in April Bearded (+1%). Again, the dwarf *Rht1 + 2* (5%–16%) and the extreme dwarfs *Rht3* (5%–16%) and *Rht2 + 3* (4%–11%) reduced grain area to a higher extent than semi‐dwarf *Rht1* (5%–8%) and *Rht2* (1%–7%). With the exception of Bersee (*Rht3* and *Rht2 + 3*), the reduction of grain area was significant.

Thousand kernel weight was strongly correlated (*p* ≤ 0.001) to grain area (*r* = 0.91), grain width (*r* = 0.93) and grain length (*r* = 0.73) confirming that smaller kernels caused the lower thousand kernel weight.

The reduction of thousand kernel weight is in agreement with previous studies (Börner et al. 1993; Nagel et al. 2013; Schierenbeck et al. 2024). Nagel et al. (2013) further observed that *Rht* alleles reduced kernel width and area, but not kernel length, which agrees with our study. Schierenbeck et al. (2024) monitored grains per square meter from our samples harvested in 2021 and 2022 and here 2023 as well as the mean of three years is summarized (Table S3). Even though the thousand kernel weight decreased due to *Rht* alleles, the grains per square meter increased remarkably (*Rht1*: +27%, *Rht2*: +36%, *Rht3*: +29% and *Rht1 + 2*: +38%) with the exception of *Rht2 + 3* (+3%). According to Börner et al. (1993), Schierenbeck et al. (2024) and our current study, the increase in grain number was sufficient to compensate for the reduction of thousand kernel weight, resulting in higher grain yields especially in NILs carrying alleles *Rht1* (+15%), *Rht2* (+22%) and *Rht1 + 2* (+6%). However, the extreme dwarf *Rht2 + 3* reduced grain yield by −21% and that of *Rht3* was stable (−2%) compared to tall *rht*. This statement is in agreement with Casebow et al. (2016), who observed that very tall plants (*rht*) and very short plants (*Rht3* and *Rht2 + 3*) had lower grain yields compared to medium tall plants (*Rht1*, *Rht2*, and *Rht1 + 2*) from Maris Huntsman and Maris Widgeon. Previous studies have indicated that genotypes carrying the semi‐dwarf *Rht1* and *Rht2* genes exhibit a higher reproductive sink strength resulting in a higher number of productive florets, grains per spike, and consequently, elevated grain number per square meter and grain yield (Sherman et al. 2014; Slafer et al. 2022; Tang et al. 2020).

Although plant height and grain morphology are not directly related to gluten composition, they are closely associated with crude protein content (see Section 3.3). Therefore, these traits were included to provide a more comprehensive understanding of how *Rht* alleles affect protein accumulation and composition.

### 3.3 Crude Protein Content

**FIGURE 3:**
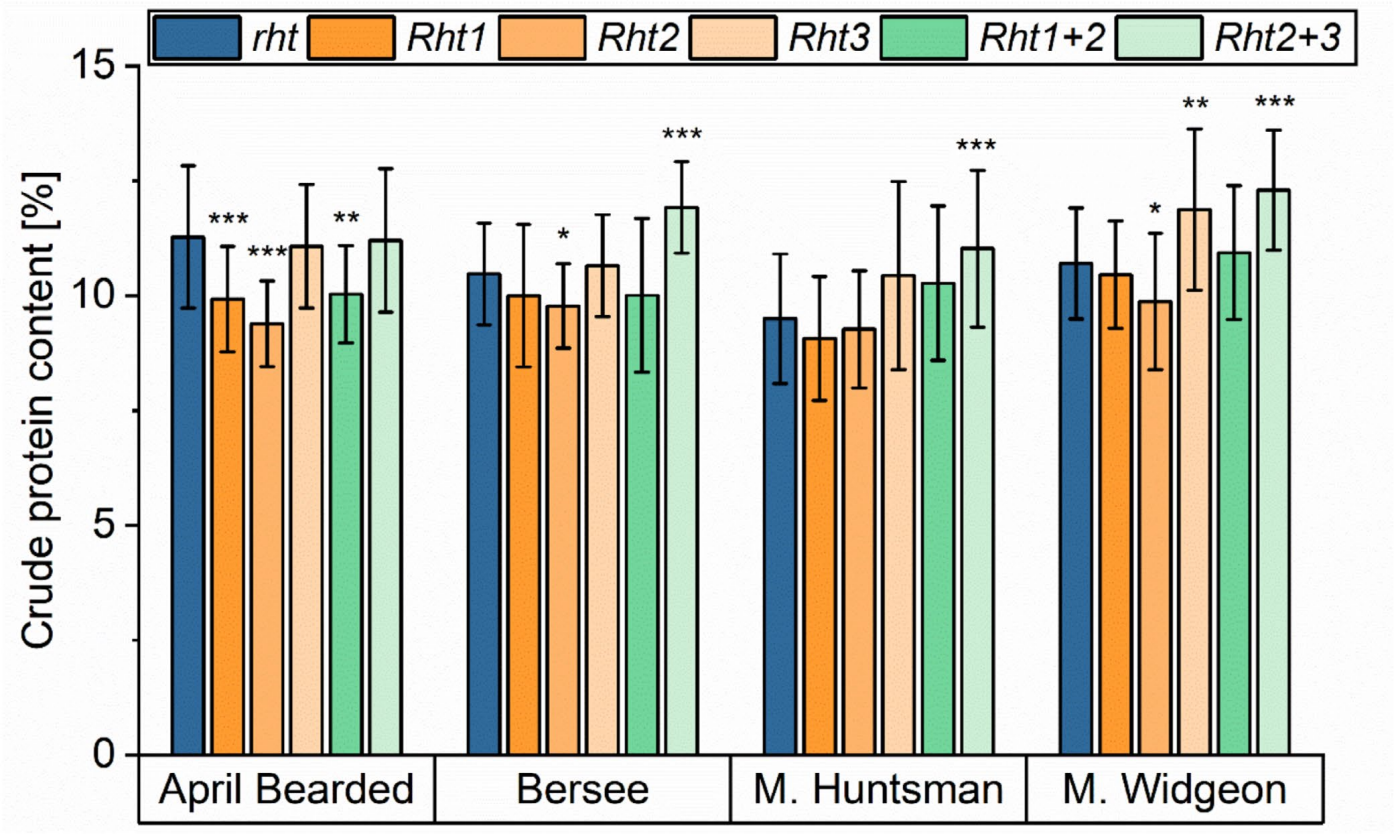
Crude protein content of NILs of the four genotypes April Bearded, Bersee, Maris (M.) Huntsman and Maris (M.) Widgeon with different *Rht* alleles. *Rht1* (semi‐dwarf), *Rht2* (semi‐dwarf), *Rht3* (extreme dwarf), *Rht1 + 2* (dwarf), and *Rht2 + 3* (extreme dwarf) are compared to wild‐type *rht* (tall). Mean of three years and three biological replicates per year (2021, 2022, and 2023; *n* = 9). Significant differences to the control are marked with asterisks (*t*‐test, *p ≤ 0.05; **p < 0.01; ***p < 0.001).

The mean crude protein content varied between 9.1% (*Rht1* of Maris Huntsman) and 12.3% (*Rht2 + 3* of Maris Widgeon) within the sample set (Figure 3, Table S3) and was lower for 2022 (7.4%–10.9%) than for 2021 (9.5%–13.4%) and 2023 (10.3%–13.4%) (Figure S3). In general, Maris Huntsman was characterized by a lower protein content in all three years compared to the other three genotypes.

The semi‐dwarf *Rht1* (−2% to −5%) and *Rht2* (−2% to −8%) reduced the crude protein content in all genotypes, while the dwarf *Rht1 + 2* mostly led to similar contents compared to *rht* (tall). With the exception of Maris Huntsman, the decrease in crude protein content was significant for semi‐dwarf *Rht2* compared to the tall wild‐type (*rht*). Further, the decrease was significant for April Bearded *Rht1* compared to *rht*. The extreme dwarf *Rht3* tended to slightly increase the crude protein content or it was the same as for the wild‐type (*rht*). With the exception of April Bearded, the extreme dwarf *Rht2 + 3* increased the crude protein content (14%–16%) compared to *rht* (tall) significantly. This significant increase was not only observed in the mean of three years, but also in each year.

Casebow et al. (2016) showed that there was a quadratic relationship between crude protein content and plant height, which was also confirmed in our study (Figure S4). This means that the crude protein content can be high in tall plants, but it is also high in short plants. Plants with medium plant height had low crude protein contents. Pinthus and Gale (1990) revealed similar results in NILs of Bersee, Maris Huntsman, and Maris Widgeon, because semi‐dwarf *Rht1* and *Rht2* had a lower protein content compared to tall *rht*, but the extreme dwarf *Rht3* showed almost the same protein content compared to the wild‐type.

The crude protein content was negatively correlated (*p* ≤ 0.001) to thousand kernel weight (*r* = −0.38), grain area (*r* = −0.27), grain width (*r* = −0.26) and grain length (*r* = −0.22, *p* ≤ 0.01). This shows that the smaller kernels from NILs with *Rht3*, *Rht1 + 2*, and *Rht2 + 3* accumulated more proteins compared to the wild‐type (*rht*). It has to be mentioned that the higher protein content might also be due to incomplete starch filling, which is especially true for dwarf *Rht1 + 2* and extreme dwarfs *Rht3* and *Rht2 + 3*, and to a lower extent for semi‐dwarfs *Rht1* and *Rht2* (Figure S2).

In a broader context, it is well known that there is the phenomenon of increasing yield combined with decreasing plant height and decreasing crude protein. Cultivars that were grown after the Green Revolution had a lower protein content, a lower plant height, and a higher yield compared to cultivars before the introduction of *Rht* alleles due to dilution effects (Laidig et al. 2017; Maeoka et al. 2020; Pronin et al. 2020; Rakszegi et al. 2008). Our study demonstrated that extreme dwarf alleles (*Rht3* and *Rht2 + 3*) can compensate for the effect of decreased crude protein content in contrast to semi‐dwarf (*Rht1* and *Rht2*) and dwarf (*Rht1 + 2*) alleles.

In general, the protein content of 10.5% (mean of all samples) was very low due to no fertilization, and one might conclude that these samples are not suitable for baking. First, it was shown that *Rht1*, *Rht2*, and *Rht3* have a higher nitrogen use efficiency than tall *rht* (Ukozehasi et al. 2022). To minimize the influence of fertilization and isolate the impact of alleles, the samples were not fertilized. Second, not only does the overall protein content determine the baking quality, but the gluten protein composition much more, and thus, the composition was characterized by Osborne fractionation and RP‐HPLC analysis.

### 3.4 Absolute Protein Composition

The Osborne fractions albumins/globulins, gliadins, and glutenins were extracted and analyzed by RP‐HPLC (Table S4). The mean albumin/globulin content ranged between 21.2 mg/g and 28.0 mg/g (Figure S5); the mean gliadin content ranged between 42.8 mg/g and 70.9 mg/g (Figure S6) and the mean glutenin content ranged between 10.7 mg/g and 23.2 mg/g (Figure S7).

The semi‐dwarfs *Rht1* and *Rht2* did not significantly increase or decrease the albumin/globulin content, with the exception of *Rht2* in April Bearded (−6%). The dwarf *Rht1 + 2* only increased the content in Maris Huntsman (8%) and Maris Widgeon (12%). The extreme dwarfs *Rht3* (8%–13%) and *Rht2 + 3* (11%–15%) significantly increased the albumin/globulin content compared to the tall wild‐type. This increase in albumin/globulin content in extreme dwarfs may reflect a shift in nitrogen allocation or altered grain development.

Due to the high variation of the crude protein content in the three harvest years, the gliadin content in all samples of 2022 differed greatly compared to those of 2021 and 2023. The semi‐dwarfs *Rht1* (−5% to −21%) and *Rht2* (−6% to −29%) either decreased the mean gliadin content, or the content was similar to the tall wild‐type (*rht*). With the exception of April Bearded (22%), the dwarf *Rht1 + 2* had no effect on gliadin content. The extreme dwarf *Rht3* either increased the content (9%–24%) or the content was similar compared to *rht*. Except for April Bearded, the extreme dwarf *Rht2 + 3* increased the gliadin content in all genotypes (24%–34%) compared to the wild‐type (*rht*). These results suggest that extreme dwarfing alleles may promote gliadin accumulation, potentially due to altered grain filling.

The mean glutenin content decreased due to *Rht* alleles, even though the decrease was not significant for all genotypes and alleles, but the decreasing trend was unambiguously observable. The decrease was dependent on the *Rht* allele and on the genotype, and it was not possible to identify *Rht* alleles that decreased the content most effectively: Semi‐dwarf *Rht1* (−9% to −26%) and *Rht2* (−13% to −37%), dwarf *Rht1 + 2* (−5% to −39%) and extreme dwarf *Rht3* (−9% to −36%) and *Rht2 + 3* (−14% to −30%). The consistent reduction of glutenin content in all *Rht* alleles could be a general compromise between plant growth and the synthesis of polymeric storage proteins. Further, the reduction might affect dough strength and elasticity.

The sum of gliadins and glutenins refers to the gluten proteins. The semi‐dwarf *Rht1* and *Rht2* significantly decreased the gluten content in April Bearded (−18% and −28%) and Bersee (−12% and −9%) and the gluten content was also lower in Maris Huntsman (−7% and −4%) and Maris Widgeon (−9% and −16%) compared to the respective *rht* (Figure S8A). With the exception of Bersee (−10%), the dwarf *Rht1 + 2* had no effect on the gluten content. Further, no effect was observed for extreme dwarf *Rht3*. However, with the exception of April Bearded, extreme dwarf *Rht2 + 3* increased the gluten content (11%–19%) compared to the wild‐type (*rht*) significantly. These findings indicate that while semi‐dwarfing alleles tend to reduce total gluten content, the extreme dwarf *Rht2 + 3* may enhance gluten accumulation.

### 3.5 Relative Protein Composition

**FIGURE 4:**
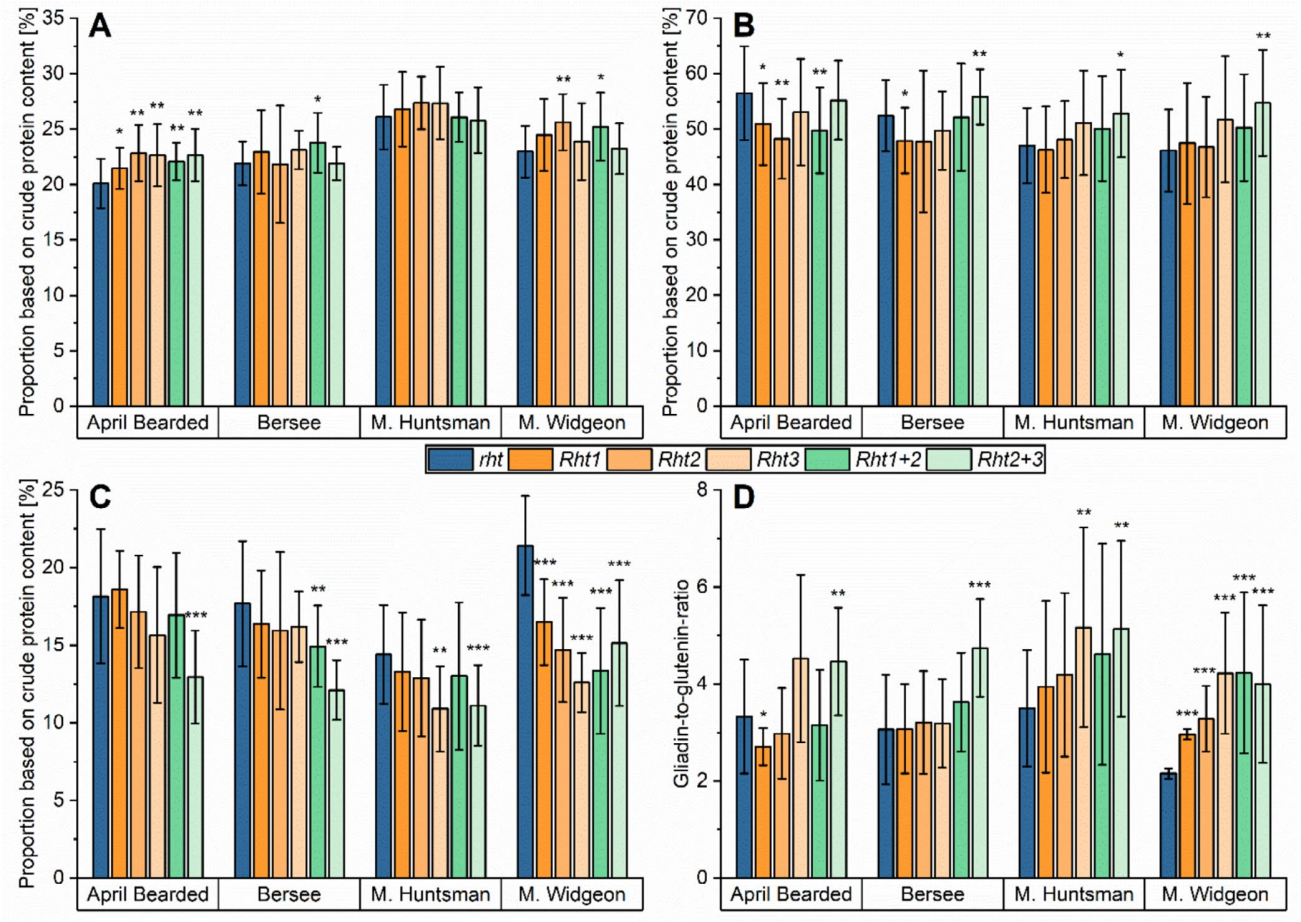
Proportion of Osborne fractions based on crude protein content and gliadin‐to‐glutenin ratio of NILs of the four genotypes April Bearded, Bersee, Maris (M.) Huntsman and Maris (M.) Widgeon with different *Rht* alleles. *Rht1* (semi‐dwarf), *Rht2* (semi‐dwarf), *Rht3* (extreme dwarf), *Rht1 + 2* (dwarf), and *Rht2 + 3* (extreme dwarf) are compared to wild‐type *rht* (tall). (A) Albumins/globulins; (B) gliadins; (C) glutenins; (D) ratio between gliadins and glutenins. Mean of 3 years and three biological replicates per year (2021, 2022, and 2023; *n* = 9). Significant differences to the control are marked with asterisks (*t*‐test, *p ≤ 0.05; **p < 0.01; ***p < 0.001).

To compensate for varying crude protein contents, especially in 2022 compared to 2021 and 2023, the contents of Osborne fractions were expressed as proportions based on crude protein content (Figure 4, Table S5). Nevertheless, high variations were observed between the three years, as demonstrated by the large standard deviations of the mean.

The proportions of albumins/globulins were not clearly affected by *Rht* alleles, and the effect was dependent on the genotype (Figure 4A). For Maris Huntsman, no significant differences were observed. In Bersee, dwarf *Rht1 + 2* (9%) and in Maris Widgeon, *Rht2* (12%) and *Rht1 + 2* (10%) increased the proportion significantly. Only in April Bearded did all *Rht* alleles have a higher proportion (7%–14%) than the wild‐type (*rht*).

The proportion of gliadins was less affected by the genotype compared to the proportion of albumins/globulins (Figure 4B). The semi‐dwarf *Rht1* and *Rht2* had the same gliadin proportion in Maris Huntsman and Maris Widgeon compared to the tall wild‐type (*rht*), but those alleles led to lower gliadin proportions in April Bearded (−10% and −15%) and Bersee (−8% and −9%). With the exception of April Bearded (−12%), dwarf *Rht1 + 2* had the same gliadin proportion as the tall *rht*, and extreme dwarf *Rht3* led to no changes in all genotypes. Except for April Bearded, the extreme dwarf *Rht2 + 3* increased the proportion of gliadins (7%–19%) significantly.

The effect of *Rht* alleles on the glutenin proportion was even clearer (Figure 4C), as a decreasing trend for all genotypes was observed, but the decrease was not significant for all alleles. Only in Maris Widgeon did the semi‐dwarf *Rht1* (−23%) and *Rht2* (−31%) decrease the mean proportion of glutenins significantly, but in the other genotypes, the proportion was lower or similar (2% to −8% and −5% to 11%, respectively) compared to the tall wild‐type (*rht*). The decrease of dwarf *Rht1 + 2* was only significant in Bersee (−16%) and Maris Widgeon (−38%; April Bearded: −7%; Maris Huntsman: −10%) and that of extreme dwarf *Rht3* in Maris Huntsman (−24%) and Maris Widgeon (−41%; April Bearded: −14%; Bersee: −10%). Especially, extreme dwarf *Rht2 + 3* decreased the proportion of glutenins (−23% to −31%) to a high extent in all genotypes.

The proportion of gluten proteins (as sum of gliadins and glutenins) was almost not affected by *Rht* alleles, with some significant exceptions (Figure S8B). All alleles of Maris Huntsman and Maris Widgeon had no significant difference in the mean gluten proportion compared to the tall wild‐type (*rht*). The gluten proportion was therefore only significantly affected by *Rht2* (−12%), *Rht1 + 2* (−11%) and *Rht2 + 3* (−9%) in April Bearded and by *Rht1* in Bersee (−8%).

The effect of *Rht* genes on gluten protein composition is displayed in the gliadin‐to‐glutenin ratio, where similar trends were seen for all genotypes, but this was not true for all alleles (Figure 4D, Figure S9). The effect of the semi‐dwarf *Rht1* and *Rht2* was not clear. In Bersee and Maris Huntsman, no significant difference was detected compared to the tall *rht*. However, in Maris Widgeon, these alleles led to significantly higher ratios (38% and 52%), but in April Bearded, *Rht1* showed a lower ratio (−19%) compared to *rht*. Only in Maris Widgeon, the dwarf *Rht1 + 2* led to a significantly higher ratio (96%). The clearest effect was observed for the extreme dwarf *Rht2 + 3* in all genotypes, because this allele increased the gliadin‐to‐glutenin ratio by 34% up to 86% compared to the tall wild‐type (*rht*).

The semi‐dwarf *Rht1* and *Rht2* and the dwarf *Rht1 + 2* are present in more than 70% of wheat cultivars used today (Evans 1998; Wuerschum et al. 2017) and thus, these alleles play a more important role than the extreme dwarf *Rht3* and *Rht2 + 3*. We showed that the semi‐dwarf and dwarf alleles had a lower effect on the gluten protein composition compared to the extreme dwarf alleles. The semi‐dwarf and dwarf alleles only led to a lower increase in the gliadin‐to‐glutenin ratio compared to the extreme dwarf alleles. Further, the semi‐dwarf and dwarf alleles were responsible for a decreased glutenin content to a lower extent than the extreme dwarf alleles. It is well known that cultivars that were commonly cultivated after the Green Revolution have higher glutenin contents and lower gliadin‐to‐glutenin ratios compared to cultivars before (Pronin et al. 2020). Based on our results, we conclude that the introduction of the *Rht* genes (here: *Rht1*, *Rht2* and *Rht1 + 2*) was not responsible for the higher glutenin content and lower gliadin‐to‐glutenin ratio of modern wheat cultivars. Last, the extreme dwarf *Rht3* and *Rht2 + 3* genes are not recommended to be introduced in wheat due to their decreasing effect on glutenins.

### 3.6 Relative Proportion of Gliadin Types and Glutenin Subunits

**FIGURE 5:**
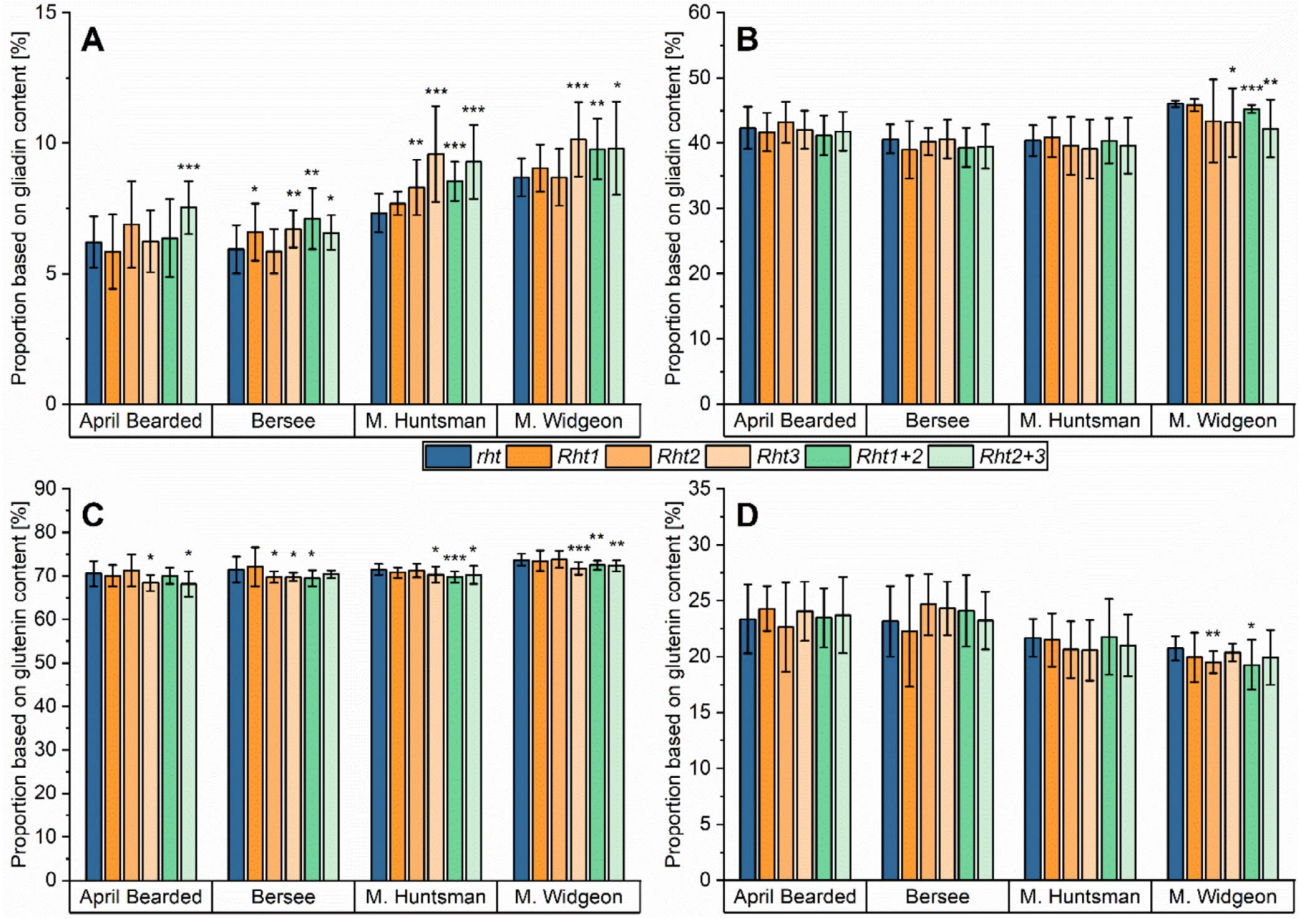
Proportion of gliadin types based on gliadin content and glutenin subunits based on glutenin content of NILs of the four genotypes April Bearded, Bersee, Maris (M.) Huntsman, and Maris (M.) Widgeon with different *Rht* alleles. *Rht1* (semi‐dwarf), *Rht2* (semi‐dwarf), *Rht3* (extreme dwarf), *Rht1 + 2* (dwarf), and *Rht2 + 3* (extreme dwarf) are compared to wild‐type *rht* (tall). (A) ω5‐gliadins; (B) α‐gliadins; (C) low‐molecular‐weight glutenin subunits (LMW‐GS); (D) high‐molecular‐weight glutenin subunits (HMW‐GS). Mean of three years and three biological replicates per year (2021, 2022 and 2023; *n* = 9). Significant differences to the control are marked with asterisks (*t*‐test, *p ≤ 0.05; **p < 0.01; ***p < 0.001).

At first glance, it seemed that *Rht* alleles had no significant effect on the proportion of gliadin types (ω5‐, ω1,2‐, α‐ and γ‐gliadins) or of glutenin subunits (HMW‐GS and LMW‐GS) in all genotypes (Figure 5, Figure S10). Overall, the tall *rht* and the semi‐dwarf *Rht1* and *Rht2* had similar proportions of gliadin types and glutenin subunits in all genotypes, and no significant difference was observed. However, the clearest effect was observed for ω5‐gliadins (Figure 5A) and LMW‐GS (Figure 5C) for dwarf *Rht1 + 2* and extreme dwarfs *Rht3* and *Rht2 + 3*. The proportion of ω5‐gliadins was significantly higher compared to the wild‐type (*rht*) in all genotypes with extreme dwarf *Rht2 + 3* (11%–27%) and in all genotypes except April Bearded with *Rht1 + 2* (12%–20%) and *Rht3* (13%–31%). In contrast, no significant differences were observed for ω1,2‐gliadins (Figure S10A) and only a few differences for α‐gliadins (Figure 5B) and for γ‐gliadins (Figure S10B). The proportion of LMW‐GS was significantly lower in all genotypes having extreme dwarf *Rht2 + 3* except in Bersee, in all genotypes with dwarf *Rht1 + 2* except in April Bearded, and in all genotypes with extreme dwarf *Rht3* compared to *rht* (tall).

To our best knowledge, this is the first study that conducted Osborne fractionation on NILs with different *Rht* alleles. Only one study extracted wheat proteins using SDS to extract first albumin/globulins and gliadins, and after sonication also the glutenins from NILs with semi‐dwarf *Rht1*, *Rht2*, and *Rht8* (Jobson et al. 2020). Quantitation was done by size exclusion chromatography. Only small differences in the gluten protein composition were observed, but interestingly, the proportion of HMW‐GS was higher in genotypes carrying semi‐dwarf alleles compared to the wild‐type (*rht*). These findings are in contrast to our study, because we showed that the glutenin content and the proportion of glutenins based on crude protein were lower because of *Rht* alleles. Furthermore, the proportion of HMW‐GS was almost the same in all samples and thus, due to the decrease of glutenins, also HMW‐GS were less present in genotypes with *Rht* genes compared to the wild‐type (*rht*). The discrepancy to literature is likely due to the use of different analytical methods and the analysis of different NILs.

The most immunoreactive gluten proteins that trigger celiac disease and wheat allergy are α‐ and ω‐gliadins as well as γ‐gliadins, LMW‐GS, and HMW‐GS to a lesser extent. The so‐called 33‐mer peptide is one of the most immunoreactive peptides (Qiao et al. 2004) and is present in α‐gliadins. Based on the positive correlation between 33‐mer peptide and α‐gliadin content (Schalk, Lang, et al. 2017), the α‐gliadin content can be used to conclude on the immunoreactive potential with regard to the 33‐mer peptide. The α‐gliadin content was lower due to semi‐dwarf *Rht1* (−4% to −23%) and *Rht2* (−8% to −28%) with the exception of *Rht1* in Maris Widgeon and *Rht2* in Maris Huntsman, which were similar compared to the tall *rht*. Thus, no increased immunoreactive potential with regard to the 33‐mer peptide is expected due to the semi‐dwarfing genes. However, the effect of dwarf *Rht1 + 2* depended on the genotype, as the α‐gliadin content was higher in Maris Huntsman (17%) and Maris Widgeon (10%), similar in Bersee, and lower in April Bearded (−25%). In contrast, the effect of the extreme dwarfing genes was clearer because the α‐gliadin content was higher due to *Rht3* (11%–18%) with the exception of Bersee (1%) and due to *Rht2 + 3* (21%–28%) with the exception of April Bearded (−6%) compared to the tall wild‐type (*rht*). However, it has to be stated that both protein and gliadin content were also higher. Thus, the relative proportion of α‐gliadins was similar due to different *Rht* genes compared to *rht* (tall). To conclude, no increase in the immunoreactive potential is expected with regard to the 33‐mer peptide due to *Rht* genes.

The main triggers of WDEIA are ω5‐gliadins and HMW‐GS (Gabler et al. 2021; Kucek et al. 2015). The semi‐dwarfs *Rht1* (−4% to −27%) and *Rht2* (−8% to −21%) had a decreasing effect on the content of ω5‐gliadins with the exception of *Rht1* in Maris Huntsman (4%) and *Rht2* in Maris Huntsman (11%). Only for April Bearded (−22%), a decrease of ω5‐gliadins was found for *Rht1 + 2* compared to the tall wild‐type, but for all other genotypes an increase was reported (17%–34%). The increase was even clearer for the extreme dwarfs *Rht3* (14%–49%) and *Rht*2 + 3 (15%–60%). One might conclude that the introduction of the semi‐dwarfing genes did not lead to an increase in the immunoreactive potential with regard to ω5‐gliadin content, but the extreme dwarfing genes might have an increasing effect on the immunoreactive potential. However, further analysis using mass spectrometry (Norwig et al. 2024; Pronin et al. 2021; Schalk, Lang, et al. 2017) or immunological methods (Gabler et al. 2021; Lee et al. 2022) is required.

Cultivars that were usually grown before and after the Green Revolution contained almost the same amount of immunoreactive peptides (Malalgoda et al. 2018; Pronin et al. 2021) or the content was even lower in old cultivars compared to modern ones (Schalk, Lang, et al. 2017). Combining these results, it can be concluded that breeding did not increase the immunoreactive potential of wheat. Nevertheless, there is currently a lack of data characterizing the effect of *Rht* genes on the immunoreactive potential of wheat, especially using NILs more in detail. Further, alternative *Rht* genes such as *Rht8*, *Rht12*, or *Rht24* should be taken into account, even though they are used to a lesser extent for breeding purposes, as it is true for *Rht3*.

### 3.7 Influence of Genotype, Allele and Environment

**FIGURE 6:**
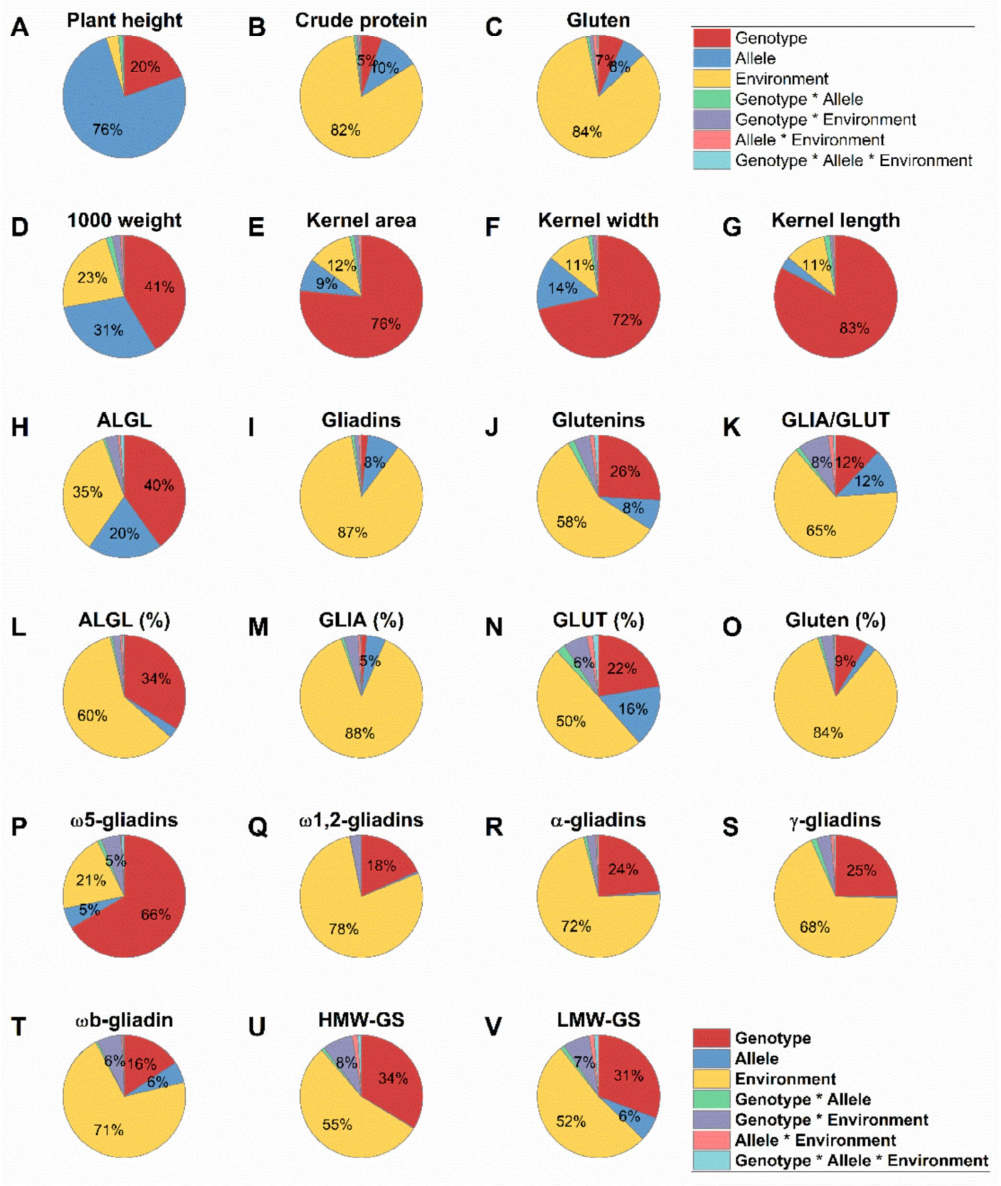
Influence of genotype, allele, and environment on plant height, crude protein content, kernel morphology, and Osborne fractions (three‐way ANOVA). (A) Plant height; (B) crude protein content; (C) gluten content as sum of gliadins and glutenins; (D) thousand kernel weight; (E) grain area; (F) grain length; (G) grain width; (H) albumins/globulins (ALGL); (I) gliadins; (J) glutenins; (K) gliadin‐to‐glutenin ratio (GLIA/GLUT). (L–O) Proportion of Osborne fractions based on crude protein content: (L) ALGL; (M) gliadins (GLIA); (N) glutenins (GLUT); (O) gluten. (P–S) Proportion of gliadin types based on gliadin content: (P) ω5‐Gliadins; (Q) ω1,2‐gliadins; (R) α‐gliadins; (S) γ‐gliadins. (T–V) Proportion of glutenin subunits based on crude protein content: (T) ωb‐gliadins; (U) high‐molecular‐weight glutenin subunits (HMW‐GS); (V) low‐molecular‐weight glutenin subunits (LMW‐GS). *F*‐values lower than 5% are not displayed. Detailed *F*‐values and significance levels are summarized in Table S6.

A three‐way ANOVA displays the effect of genotype (April Bearded, Bersee, Maris Huntsman, Maris Widgeon), *Rht* allele (*rht*, *Rht1*, *Rht2*, *Rht3*, *Rht1 + 2* and *Rht2 + 3*) and environment (harvested in 2021, 2022 and 2023) on the parameters analyzed (Figure 6, Table S6). Only plant height was predominantly affected by *Rht* alleles (76%) and to a very low extent by the environment (3%), which shows a very high genetic stability. The effect of *Rht* allele was only higher than or equal to 10% for crude protein content (10%), thousand kernel weight (31%), kernel width (14%), albumin/globulin content (20%), gliadin‐to‐glutenin ratio (12%) and proportion of glutenins (16%). Kernel area (76%), kernel width (72%), kernel length (83%) and proportion of ω5‐gliadins (66%) were most affected by genotype. With the exception of albumin/globulin content (35%) and proportion of ω5‐gliadins (21%), all results related to protein composition were most affected by the environment (≥ 50%) showing a low genetic stability. Furthermore, the impact of genotype was even higher than that of allele for gluten content, albumin/globulin content, glutenin content, gliadin‐to‐glutenin ratio, proportion of albumins/globulins, proportion of gliadins, proportion of glutenins, proportion of gluten, and proportion of ω1,2‐ α‐, and γ‐gliadins.

Interactions between two or three factors were mostly negligible compared to the effect of genotype, allele, and environment, but the majority of interactions were still significant (Table S6). Only the interaction between genotype and environment had an influence of more than 5% on gliadin‐to‐glutenin ratio (8%), proportions of glutenins (6%), those of ω5‐gliadins (5%), those of HMW‐GS (8%) and LMW‐GS (7%).

The climatic conditions varied greatly between the three years (https://wetter.ipk‐gatersleben.de). The mean annual temperature was 9.6°C in 2021, 10.8°C in 2022, and 11.0°C in 2023. The cumulative precipitation was almost twice as much (782 mm) in 2023 compared to 2021 (437 mm) and 2022 (394 mm). To sum up, 2021 was cool and dry, 2022 was warm and dry, and 2023 was warm and wet. These differences are also reflected in the crude protein content, which was lowest in 2022 (mean of all samples 8.9%) followed by 2021 (10.8%) and by 2023 (11.8%). The same order was obtained for albumin/globulin content (lowest in 2022: 23.2 mg/g and followed by 2021: 24.7 mg/g and by 2023: 26.0 mg/g) and gliadin content (lowest in 2022: 37.1 mg/g, followed by 2021: 58.5 mg/g and by 2023: 66.0 mg/g). In contrast, the glutenin content was lowest in 2021 (13.4 mg/g) followed by 2022 (14.4 mg/g) and 2023 (19.5 mg/g).

Grain filling including protein accumulation occurs within a 4‐week period after flowering (Call, Haider, et al. 2020; Shewry et al. 2012) and the time point of flowering is dependent on environmental conditions but is usually in June in Germany (Langer et al. 2014). The flowering date of our sample set was between 7th and 9th June in 2021, between 28th and 30th May in 2022, and between 5th and 9th June in 2023. The earlier flowering date in 2022 was due to drought conditions in May.

The weather in June 2023 was relatively cool (18.8°C) and wet (111 mm) compared to June 2021 (20.0°C and 69 mm) and June 2022 (19.3°C and 28 mm) and July was almost the same in the three years (≈20°C and ≈40 mm). One might conclude that cool and wet conditions (2023) during grain filling led to higher crude protein and glutenin content than warm and wet (2021) ones or warm and dry ones (2022). Furthermore, warm and dry conditions (2022) resulted in very low gliadin content compared to cold and wet weather (2023) and warm and wet weather (2022), but at the same time, to low gliadin‐to‐glutenin ratios. The very strong influence of environmental conditions on protein composition is consistent with the literature (Call, Kapeller, et al. 2020; Filip et al. 2023; Geisslitz et al. 2019; Pronin et al. 2020; Ronga et al. 2020) and our study strongly supports previous findings. Overall, the environmental conditions have indeed a major influence on the gluten protein composition, making breeding for the “perfect” gluten composition particularly challenging.

## 4 Conclusion

The Green Revolution *Rht* genes were introduced into wheat to decrease plant height and to increase yield and harvest index, but their effect on gluten protein composition had not been systematically studied. We demonstrated that the *Rht* genes not only had an effect on crude protein content, but they also changed the gluten protein composition, and the change was dependent on the genotype and the allele. Our results showed that the semi‐dwarfing alleles *Rht1* and *Rht2*, as well as the dwarf allele *Rht1 + 2*, had minor effects on gluten protein composition. In contrast, the extreme dwarfing alleles *Rht3* and *Rht2 + 3* led to a notable reduction in glutenin content and an increase in gliadin‐to‐glutenin ratios, which may negatively affect baking quality. As only the semi‐dwarfing and dwarfing alleles are present in modern wheat cultivars, we can conclude that the introduction of the *Rht* genes alone does not explain the observed differences in gluten composition between modern and pre‐Green Revolution cultivars. Given the lower glutenin content and possible higher immunoreactive potential associated with extreme dwarfs *Rht3* and *Rht2 + 3*, these alleles may not be advisable for inclusion in future wheat breeding programs focused on baking quality and health‐related traits. Three biological replicates from three harvest years allow us to demonstrate that environmental conditions had a substantial influence on gluten protein composition, and the high number of biological replicates strengthens our findings. Future work will focus on proteomic insights to evaluate the immunoreactive potential of various *Rht* alleles more in detail.

## Author Contributions

**Sabrina Geisslitz:** conceptualization (equal), formal analysis (equal), investigation (equal), methodology (equal), visualization (equal), writing – original draft (equal). **Matías Schierenbeck:** investigation (equal), methodology (equal), resources (equal), writing – review and editing (equal). **Andreas Börner:** conceptualization (equal), methodology (equal), resources (equal), writing – review and editing (equal). **Katharina Anne Scherf:** conceptualization (equal), resources (equal), supervision (equal), writing – review and editing (equal).

## Conflicts of Interest

The authors declare no conflicts of interest.

## Supporting information


Appendix S1

Table S3

Table S4

Table S5

Table S6

## Data Availability

Data about kernel morphology, crude protein content, and Osborne fractionation are summarized in Table S4.

## References

[fsn370649-bib-0001] Boeven, P. H. G. , C. F. H. Longin , W. L. Leiser , S. Kollers , E. Ebmeyer , and T. Würschum . 2016. “Genetic Architecture of Male Floral Traits Required for Hybrid Wheat Breeding.” Theoretical and Applied Genetics 129: 2343–2357. 10.1007/s00122-016-2771-6. 27553082

[fsn370649-bib-0002] Börner, A. , A. J. Worland , J. Plaschke , E. Schumann , and C. N. Law . 1993. “Pleiotropic Effects of Genes for Reduced Height (*Rht*) and Day‐Length Insensitivity (Ppd) on Yield and Its Components for Wheat Grown in Middle Europe.” Plant Breeding 111: 204–216. 10.1111/j.1439-0523.1993.tb00631.x.

[fsn370649-bib-0003] Call, L. , E. Haider , S. D'Amico , E. Reiter , and H. Grausgruber . 2020. “Synthesis and Accumulation of Amylase‐Trypsin Inhibitors and FODMAPs in Bread Wheat (*Triticum aestivum* L.) During Grain Development.” BMC Plant Biology 21: 113. 10.21203/rs.3.rs-72372/v1. PMC7905651 33627080

[fsn370649-bib-0004] Call, L. , M. Kapeller , H. Grausgruber , E. Reiter , R. Schoenlechner , and S. D'Amico . 2020. “Effects of Species and Breeding on Wheat Protein Composition.” Journal of Cereal Science 93: 102974. 10.1016/j.jcs.2020.102974.

[fsn370649-bib-0005] Casebow, R. , C. Hadley , R. Uppal , et al. 2016. “Reduced Height (*Rht*) Alleles Affect Wheat Grain Quality.” PLoS One 11: e0156056. 10.1371/journal.pone.0156056. 27196288 PMC4873232

[fsn370649-bib-0006] Du, Y. , L. Chen , Y. Wang , et al. 2018. “The Combination of Dwarfing Genes *Rht4* and *Rht8* Reduced Plant Height, Improved Yield Traits of Rainfed Bread Wheat ( *Triticum aestivum* L.).” Field Crops Research 215: 149–155. 10.1016/j.fcr.2017.10.015.

[fsn370649-bib-0007] Evans, L. T. 1998. Feeding the Ten Billion: Plants and Population Growth. Cambridge University Press.

[fsn370649-bib-0008] Filip, E. , K. Woronko , E. Stępień , and N. Czarniecka . 2023. “An Overview of Factors Affecting the Functional Quality of Common Wheat (*Triticum aestivum* L.).” International Journal of Molecular Sciences 24: 7524. 10.3390/ijms24087524. 37108683 PMC10142556

[fsn370649-bib-0009] Flintham, J. E. , A. Börner , A. J. Worland , and M. D. Gale . 1997. “Optimizing Wheat Grain Yield: Effects of *Rht* (Gibberellin‐Insensitive) Dwarfing Genes.” Journal of Agricultural Science 128: 11–25. 10.1017/S0021859696003942.

[fsn370649-bib-0010] Gabler, A. M. , J. Gebhard , B. Eberlein , T. Biedermann , K. A. Scherf , and K. Brockow . 2021. “The Basophil Activation Test Differentiates Between Patients With Wheat‐Dependent Exercise‐Induced Anaphylaxis and Control Subjects Using Gluten and Isolated Gluten Protein Types.” Clinical and Translational Allergy 11: e12050. 10.1002/clt2.12050. 34386193 PMC8340350

[fsn370649-bib-0011] Geisslitz, S. , C. F. H. Longin , K. A. Scherf , and P. Koehler . 2019. “Comparative Study on Gluten Protein Composition of Ancient (Einkorn, Emmer and Spelt) and Modern Wheat Species (Durum and Common Wheat).” Food 8: 409. 10.3390/foods8090409. PMC6769531 31547385

[fsn370649-bib-0012] Gooding, M. J. , R. K. Uppal , M. Addisu , et al. 2012. “Reduced Height Alleles (*Rht*) and Hagberg Falling Number of Wheat.” Journal of Cereal Science 55: 305–311. 10.1016/j.jcs.2012.01.003.

[fsn370649-bib-0013] Jahn, N. , U. Konradl , K. Fleissner , S. Geisslitz , and K. A. Scherf . 2024. “Protein Composition and Bread Volume of German Common Wheat Landraces Grown Under Organic Conditions.” Current Research in Food Science 9: 100871. 10.1016/j.crfs.2024.100871. 39435451 PMC11491675

[fsn370649-bib-0014] Jobson, E. , J.‐B. Ohm , J. Martin , and M. Giroux . 2020. “ *Rht‐1* Semi‐Dwarfing Alleles Increase the Abundance of High Molecular Weight Glutenin Subunits.” Cereal Chemistry 98: 337–345. 10.1002/cche.10371.

[fsn370649-bib-0015] Kucek, L. K. , L. D. Veenstra , P. Amnuaycheewa , and M. E. Sorrells . 2015. “A Grounded Guide to Gluten: How Modern Genotypes and Processing Impact Wheat Sensitivity.” Comprehensive Reviews in Food Science and Food Safety 14: 285–302. 10.1111/1541-4337.12129. 33401796

[fsn370649-bib-0016] Kumar, S. , A. Singh , A. P. Singh , et al. 2024. “Gluten‐Related Disorders: Current Understanding, Myths, and Facts.” In Wheat Science, edited by O. P. Gupta , S. Kumar , A. Pandey , M. K. Khan , S. K. Singh , and G. P. Singh , 321–338. CRC Press.

[fsn370649-bib-0017] Laidig, F. , H.‐P. Piepho , D. Rentel , T. Drobek , U. Meyer , and A. Huesken . 2017. “Breeding Progress, Environmental Variation and Correlation of Winter Wheat Yield and Quality Traits in German Official Variety Trials and On‐Farm During 1983–2014.” Theoretical and Applied Genetics 130: 223–245. 10.1007/s00122-016-2810-3. 27796431 PMC5215243

[fsn370649-bib-0018] Landjeva, S. , V. Korzun , E. Stoimenova , B. Truberg , G. Ganeva , and A. Börner . 2008. “The Contribution of the Gibberellin‐Insensitive Semi‐Dwarfing (*Rht*) Genes to Genetic Variation in Wheat Seedling Growth in Response to Osmotic Stress.” Journal of Agricultural Science 146: 275–286. 10.1017/S0021859607007575.

[fsn370649-bib-0019] Langer, S. M. , C. F. H. Longin , and T. Würschum . 2014. “Flowering Time Control in European Winter Wheat.” Frontiers in Plant Science 5: 537. 10.3389/fpls.2014.00537. 25346745 PMC4191279

[fsn370649-bib-0020] Lee, J. , S. R. Kim , J. H. Park , et al. 2022. “Evaluation of Allergenicity on a ω‐5 Gliadin‐Deficient Cultivar in Wheat‐Dependent Exercise‐Induced Anaphylaxis.” Allergy, Asthma & Immunology Research 14: 379–392. 10.4168/aair.2022.14.4.379. PMC9293598 35837822

[fsn370649-bib-0021] Maeoka, R. E. , V. O. Sadras , I. A. Ciampitti , D. R. Diaz , A. K. Fritz , and R. P. Lollato . 2020. “Changes in the Phenotype of Winter Wheat Varieties Released Between 1920 and 2016 in Response to In‐Furrow Fertilizer: Biomass Allocation, Yield, and Grain Protein Concentration.” Frontiers in Plant Science 10: 1786. 10.3389/fpls.2019.01786. 32082347 PMC7002544

[fsn370649-bib-0022] Malalgoda, M. , S. W. Meinhardt , and S. Simsek . 2018. “Detection and Quantitation of Immunogenic Epitopes Related to Celiac Disease in Historical and Modern Hard Red Spring Wheat Cultivars.” Food Chemistry 264: 101–107. 10.1016/j.foodchem.2018.04.131. 29853353

[fsn370649-bib-0023] Nagel, M. , A.‐K. Behrens , and A. Börner . 2013. “Effects of *Rht* Dwarfing Alleles on Wheat Seed Vigour After Controlled Deterioration.” Crop and Pasture Science 64: 857–864. 10.1071/CP13041.

[fsn370649-bib-0024] Norwig, M.‐C. , S. Geisslitz , and K. A. Scherf . 2024. “Comparative Label‐Free Proteomics Study on Celiac Disease‐Active Epitopes in Common Wheat, Spelt, Durum Wheat, Emmer, and Einkorn.” Journal of Agricultural and Food Chemistry 72: 15040–15052. 10.1021/acs.jafc.4c02657. 38906536 PMC11228976

[fsn370649-bib-0025] Pinthus, M. J. , and M. D. Gale . 1990. “The Effects of ‘Gibberellin‐Insensitive’ Dwarfing Alleles in Wheat on Grain Weight and Protein Content.” Theoretical and Applied Genetics 79: 108–112. 10.1007/BF00223795. 24226128

[fsn370649-bib-0026] Pronin, D. , A. Börner , and K. A. Scherf . 2021. “Old and Modern Wheat ( *Triticum aestivum* L.) Cultivars and Their Potential to Elicit Celiac Disease.” Food Chemistry 339: 127952. 10.1016/j.foodchem.2020.127952. 33152854

[fsn370649-bib-0027] Pronin, D. , A. Börner , H. Weber , and K. A. Scherf . 2020. “Wheat ( *Triticum aestivum* L.) Breeding From 1891 to 2010 Contributed to Increasing Yield and Glutenin Contents but Decreasing Protein and Gliadin Contents.” Journal of Agricultural and Food Chemistry 68: 13247–13256. 10.1021/acs.jafc.0c02815. 32648759

[fsn370649-bib-0028] Qiao, S. W. , E. Bergseng , Ø. Molberg , et al. 2004. “Antigen Presentation to Celiac Lesion‐Derived T Cells of a 33‐Mer Gliadin Peptide Naturally Formed by Gastrointestinal Digestion.” Journal of Immunology 173: 1757–1762. 10.4049/jimmunol.173.3.1757. 15265905

[fsn370649-bib-0029] Rakszegi, M. , D. Boros , C. Kuti , L. Láng , Z. Bedő , and P. R. Shewry . 2008. “Composition and End‐Use Quality of 150 Wheat Lines Selected for the HEALTHGRAIN Diversity Screen.” Journal of Agricultural and Food Chemistry 56: 9750–9757. 10.1021/jf8009359. 18921975

[fsn370649-bib-0030] Ronga, D. , L. Laviano , M. Catellani , et al. 2020. “Influence of Environmental and Genetic Factors on Content of Toxic and Immunogenic Wheat Gluten Peptides.” European Journal of Agronomy 118: 126091. 10.1016/j.eja.2020.126091.

[fsn370649-bib-0031] Schalk, K. , C. Lang , H. Wieser , P. Koehler , and K. A. Scherf . 2017. “Quantitation of the Immunodominant 33‐Mer Peptide From α‐Gliadin in Wheat Flours by Liquid Chromatography Tandem Mass Spectrometry.” Scientific Reports 7: 45092. 10.1038/srep45092. 28327674 PMC5361186

[fsn370649-bib-0032] Schalk, K. , B. Lexhaller , P. Koehler , and K. A. Scherf . 2017. “Isolation and Characterization of Gluten Protein Types From Wheat, Rye, Barley and Oats for Use as Reference Materials.” PLoS One 12: e0172819. 10.1371/journal.pone.0172819. 28234993 PMC5325591

[fsn370649-bib-0033] Schierenbeck, M. , A. M. Alqudah , E. Lantos , E. G. Avogadro , M. R. Simón , and A. Börner . 2024. “Green Revolution Dwarfing *Rht* Genes Negatively Affected Wheat Floral Traits Related to Cross‐Pollination Efficiency.” Plant Journal 118: 1071–1085. 10.1111/tpj.16652. 38294345

[fsn370649-bib-0034] Schierenbeck, M. , A. M. Alqudah , U. Lohwasser , R. A. Tarawneh , M. R. Simón , and A. Börner . 2021. “Genetic Dissection of Grain Architecture‐Related Traits in a Winter Wheat Population.” BMC Plant Biology 21: 417. 10.1186/s12870-021-03183-3. 34507551 PMC8431894

[fsn370649-bib-0035] Schuster, C. , J. Huen , and K. A. Scherf . 2023. “Comprehensive Study on Gluten Composition and Baking Quality of Winter Wheat.” Cereal Chemistry 100: 142–155. 10.1002/cche.10606.

[fsn370649-bib-0036] Sharma, A. , S. Garg , I. Sheikh , P. Vyas , and H. S. Dhaliwal . 2020. “Effect of Wheat Grain Protein Composition on End‐Use Quality.” Journal of Food Science and Technology 57: 2771–2785. 10.1007/s13197-019-04222-6. 32624587 PMC7316921

[fsn370649-bib-0037] Sherman, J. D. , D. Nash , S. P. Lanning , et al. 2014. “Genetics of End‐Use Quality Differences Between a Modern and Historical Spring Wheat.” Crop Science 54: 1972–1980. 10.2135/cropsci2013.11.0749.

[fsn370649-bib-0038] Shewry, P. R. , R. A. C. Mitchell , P. Tosi , et al. 2012. “An Integrated Study of Grain Development of Wheat (cv. Hereward).” Journal of Cereal Science 56: 21–30. 10.1016/j.jcs.2011.11.007.

[fsn370649-bib-0039] Slafer, G. A. , M. J. Foulkes , M. P. Reynolds , et al. 2022. “A ‘Wiring Diagram’ for Sink Strength Traits Impacting Wheat Yield Potential.” Journal of Experimental Botany 74: 40–71. 10.1093/jxb/erac410. PMC9786893 36334052

[fsn370649-bib-0040] Tang, T. , T. Botwright Acuña , W. Spielmeyer , and R. A. Richards . 2020. “Effect of Gibberellin‐Sensitive Rht18 and Gibberellin‐Insensitive Rht‐D1b Dwarfing Genes on Vegetative and Reproductive Growth in Bread Wheat.” Journal of Experimental Botany 72: 445–458. 10.1093/jxb/eraa481. 33070174

[fsn370649-bib-0041] Thanhaeuser, S. M. , H. Wieser , and P. Koehler . 2014. “Correlation of Quality Parameters With the Baking Performance of Wheat Flours.” Cereal Chemistry 91: 333–341. 10.1094/CCHEM-09-13-0194-CESI.

[fsn370649-bib-0042] Ukozehasi, C. , E. S. Ober , and H. Griffiths . 2022. “The Other Mechanisms by Which the Rht Genes Improve the Harvest Index of Wheat.” Plants 11, no. 21: 2837. 10.3390/plants11212837. 36365291 PMC9658701

[fsn370649-bib-0043] van Eckert, R. , E. Berghofer , P. J. Ciclitira , et al. 2006. “Towards a New Gliadin Reference Material‐Isolation and Characterisation.” Journal of Cereal Science 43: 331–341. 10.1016/j.jcs.2005.12.009.

[fsn370649-bib-0044] Wieser, H. , S. Antes , and W. Seilmeier . 1998. “Quantitative Determination of Gluten Protein Types in Wheat Flour by Reversed‐Phase High‐Performance Liquid Chromatography.” Cereal Chemistry 75: 644–650. 10.1094/CCHEM.1998.75.5.644.

[fsn370649-bib-0045] Wieser, H. , and R. Kieffer . 2001. “Correlations of the Amount of Gluten Protein Types to the Technological Properties of Wheat Flours Determined on a Micro‐Scale.” Journal of Cereal Science 34: 19–27. 10.1006/jcrs.2000.0385.

[fsn370649-bib-0046] Wieser, H. , P. Koehler , and K. A. Scherf . 2020. “The Two Faces of Wheat.” Frontiers in Nutrition 7: 517313. 10.3389/fnut.2020.517313. 33195360 PMC7609444

[fsn370649-bib-0047] Wieser, H. , P. Koehler , and K. A. Scherf . 2023. “Chemistry of Wheat Gluten Proteins: Qualitative Composition.” Cereal Chemistry 100: 23–35. 10.1002/cche.10572.

[fsn370649-bib-0048] Wuerschum, T. , S. M. Langer , C. F. H. Longin , M. R. Tucker , and W. L. Leiser . 2017. “A Modern Green Revolution Gene for Reduced Height in Wheat.” Plant Journal 92: 892–903. 10.1111/tpj.13726. 28949040

[fsn370649-bib-0049] Youssefian, S. , E. J. M. Kirby , and M. D. Gale . 1992. “Pleiotropic Effects of the GA‐Insensitive Rht Dwarfing Genes in Wheat. 1. Effects on Development of the Ear, Stem and Leaves.” Field Crops Research 28: 179–190. 10.1016/0378-4290(92)90039-C.

