# Supplementary material for: Semi‐Dwarfing Reduced Height Genes Hardly Influenced Gluten Protein Composition While Extreme Dwarfing Genes Decreased Glutenins in Wheat
Source: Food Sci Nutr. 2025 Jul 30;13(8):e70649. doi: 10.1002/fsn3.70649 (PMC12308216; doi:10.1002/fsn3.70649)
Supplement: Supplementary file 1 — Appendix S1 [file FSN3-13-e70649-s004.pdf]

SUPPORTING INFORMATION

Semi-dwarfing reduced height genes hardly influenced gluten protein composition while extreme dwarfing genes decreased glutenins in wheat

Impact of reduced height genes on gluten

Sabrina GEISSLITZ[1,2*], Matías SCHIERENBECK[3], Andreas BÖRNER[4], Katharina Anne SCHERF[1,2,5]

[1] Department of Bioactive and Functional Food Chemistry, Institute of Applied Biosciences, Karlsruhe Institute of Technology (KIT), Karlsruhe, Germany

[2] Leibniz Institute for Food Systems Biology at the Technical University of Munich, Freising, Germany

[3] Physiology and Cell Biology Department, Leibniz Institute of Plant Genetics and Crop Plant Research (IPK), Corrensstr. 3, 06466 Seeland/OT Gatersleben, Germany

[4] Genebank Department, Leibniz Institute of Plant Genetics and Crop Plant Research (IPK), Corrensstr. 3, 06466 Seeland/OT Gatersleben, Germany

[5] Technical University of Munich, TUM School of Life Sciences, Professorship of Food Biopolymer Systems, Freising, Germany

Correspondence: Sabrina Geisslitz, Leibniz Institute for Food Systems Biology at the Technical University of Munich, Freising, Germany, Email: s.geisslitz.leibniz-lsb@tum.de

Abbreviations: ALGL, albumins/globulins; ANOVA, analysis of variance; GLIA, gliadins; GLIA/GLUT, gliadin-to-glutenin-ratio; GLUT, glutenins; HMW-GS, high-molecular-weight glutenin subunits; LMW-GS, low-molecular-weight glutenin subunits; NILs, near isogenic lines; *Rht*, reduced height

Table S1: Average values of phosphorus (P), potassium (K), magnesium (Mg), nitrate nitrogen (NO_3-N) and ammonia nitrogen (NH_4-N) in mg/100g of soil analyzed in early spring (February/March) of each growing season.

Year	P	K	Mg	NO_3-N	NH_4-N
2021	12.8	16.8	11.7	0.330	0
2022	14.6	14.8	12.6	0.622	0
2023	13.9	15.2	11.9	0.458	0

Table S2: Number of data points per sample (e.g., for April Bearded *rht*) depending on the method considering the number of harvest years, biological and technical replicates.

Method	Harvest years	Biological replicates	Technical replicates	Number of data per sample
Kernel morphology	3	3	1	9
Crude protein (Dumas)	3	3	3	27
Osborne fractionation	3	3	2	18

Table S3: Separate Excel file. Plant height, thousand kernel weight, grain area, grain width, grain length and crude protein content of *rht* (tall), *Rht1* (semi-dwarf), *Rht2* (semi-dwarf), *Rht3* (extreme-dwarf), *Rht1+2* (dwarf) and *Rht2+3* (extreme-dwarf) of April Bearded, Bersee, Maris Huntsman and Maris Widgeon from the three harvest years A, 2021, B, 2022 and C, 2023, and D, mean of the three years. Std., standard deviation.

Table S4: Separate Excel file. Albumins/globulins, gliadins and glutenins extracted by the modified Osborne fractionation and quantitated by RP-HPLC. A, 2021, B, 2022 and C, 2023, and D, mean of the three years of *rht* (tall), *Rht1* (semi-dwarf), *Rht2* (semi-dwarf), *Rht3* (extreme dwarf), *Rht1+2* (dwarf) and *Rht2+3* extreme-dwarf) of April Bearded, Bersee, Maris Huntsman and Maris Widgeon. Total gliadin refers to the sum of ω5-, ω1,2-, α- and γ-gliadin subunits. Total glutenin types refers to the sum of ωb-gliadins, low- and high-molecular-weight glutenins subunits (LMW-GS and HMW-GS). Gluten refers to the sum of gliadins and glutenins. Sum of Osborne fractions refers to the sum of albumins/globulins, gliadins and glutenins. GLIA/GLUT, ratio between gliadins and glutenins. Insoluble proteins refer to the difference between crude protein content and sum of Osborne fractions. Std., standard deviation.

Table S5: Separate Excel file. Relative proportion based on crude protein content of albumins/globulins, gliadins and glutenins of *rht* (tall), *Rht1* (semi-dwarf), *Rht2* (semi-dwarf), *Rht3* (extreme dwarf), *Rht1+2* (dwarf) and *Rht2+3* (extreme dwarf) of April Bearded, Bersee, Maris Huntsman and Maris Widgeon from the three harvest years A, 2021, B, 2022 and C, 2023, and D, mean of the three years. Explanations see Table S4.

Table S6: Separate Excel file. Statistics of three-way analysis of variance (ANOVA) with the factors genotype, allele and environment. A, plant height; B, crude protein content; C, gluten content as sum of gliadins and glutenins; D, thousand kernel weight; E, grain area; F, grain length; G, grain width; H, albumins/globulins (ALGL); I, gliadins; J, glutenins; K, gliadin-to-glutenin-ratio (GLIA/GLUT). L-O, Proportion of Osborne fractions based on crude protein content: L, ALGL; M, gliadins (GLIA); N, glutenins (GLUT); O, gluten. P-S: Proportion of gliadin types based on gliadin content: P, ω5-Gliadins; Q, ω1,2-gliadins; R, α-gliadins; S, γ-gliadins. T-V: Proportion of glutenin subunits based on crude protein content: T, ωb-gliadins; U, high-molecular-weight glutenin-subunits (HMW-GS); V, low-molecular-weight glutenin-subunits (LMW-GS). F-values are displayed in Figure 6.

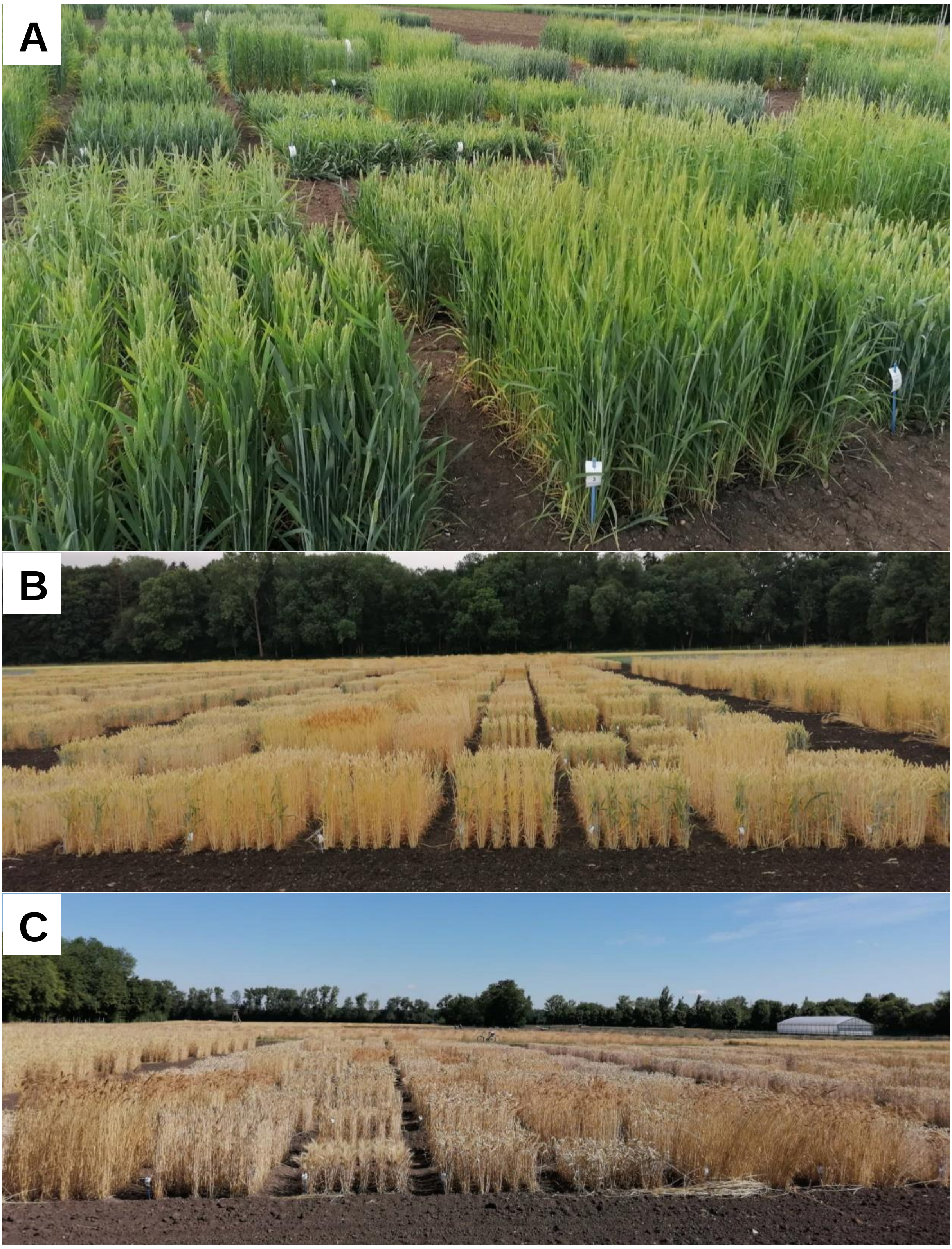

Figure S1: Field trails at different development stages of NILs (April Bearded, Bersee, Maris Huntsman and Maris Widgeon) with different *Rht* alleles in 2021. A, Flowering; B, grain filling; C, ripening.

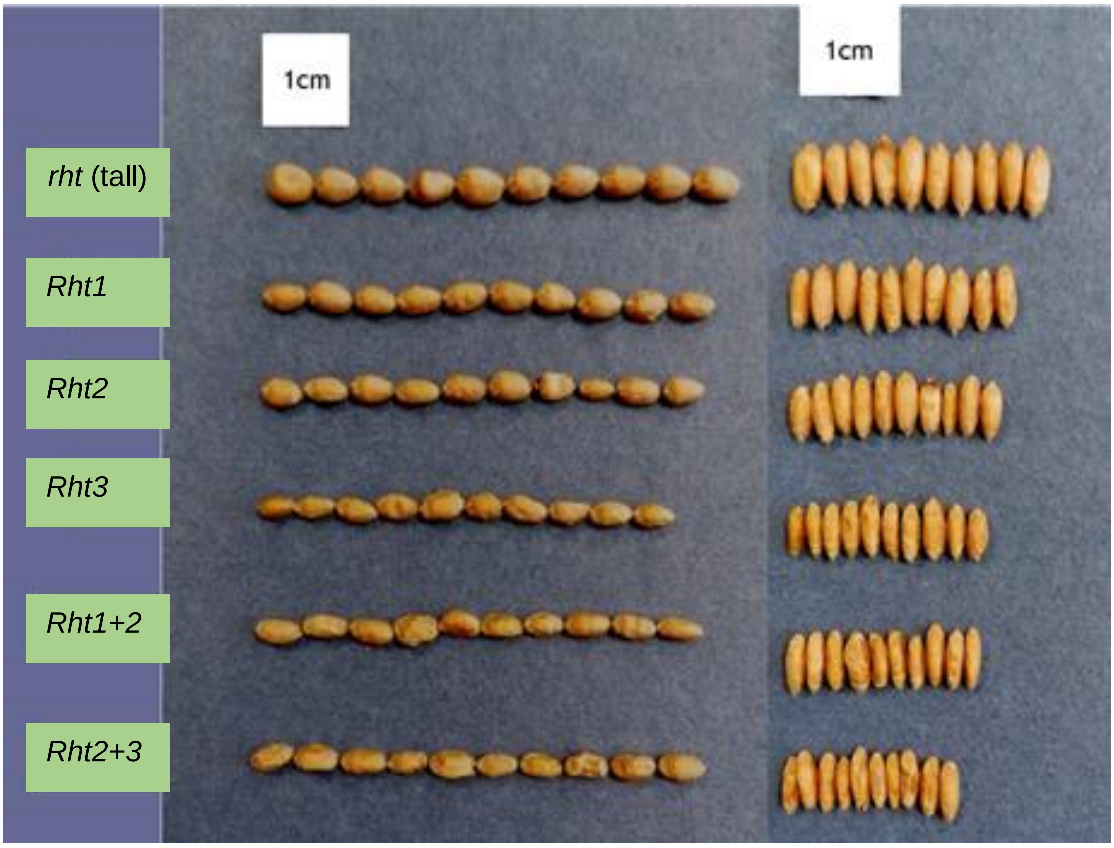

Figure S2: Length and width of ten kernels each of Maris Huntsman with different *Rht* alleles (semi-dwarf *Rht1*, semi-dwarf *Rht2*, extreme dwarf *Rht3*, dwarf *Rht1+2* and extreme dwarf *Rht2+3*) compared to tall controls (*rht*).

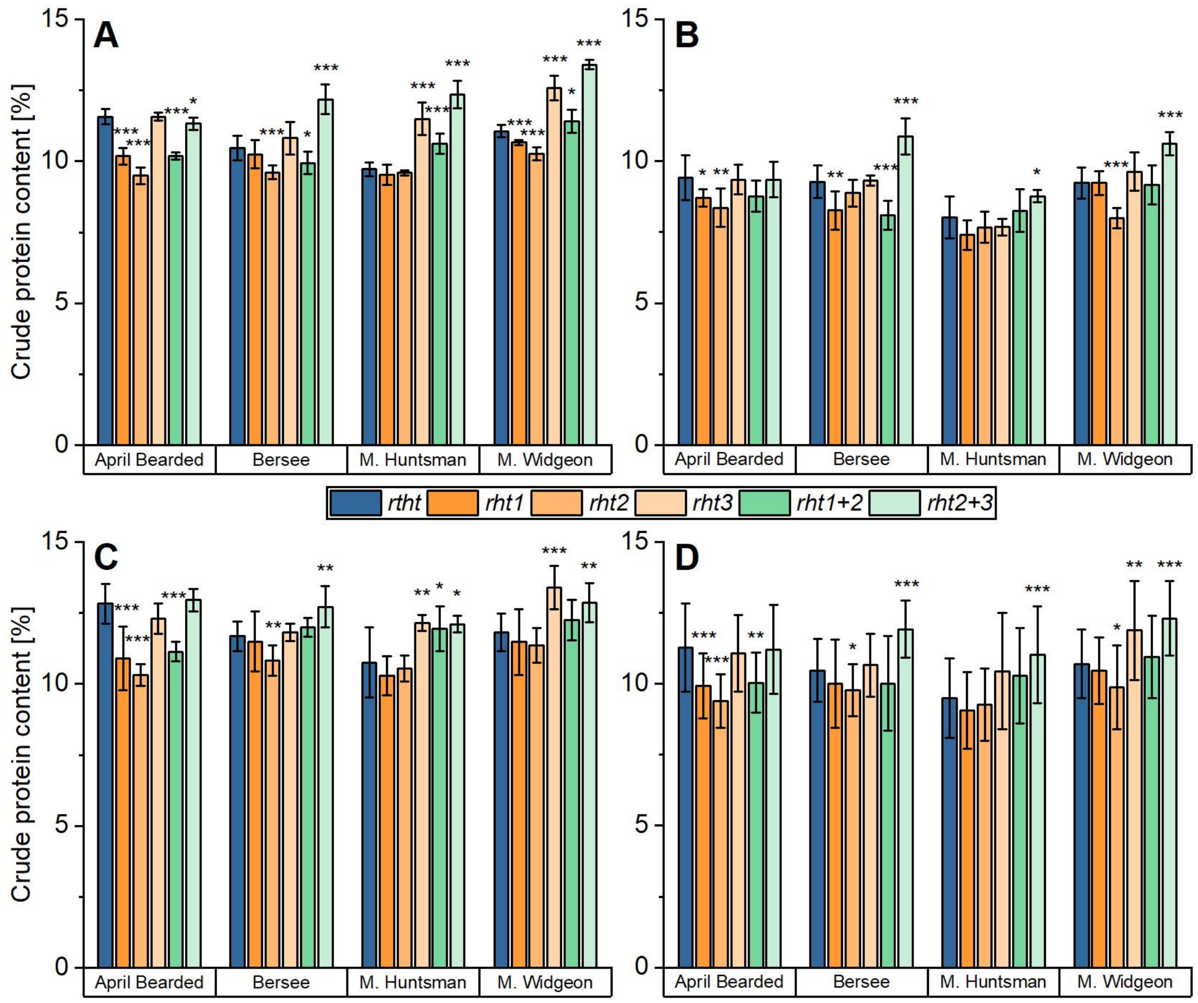

Figure S3: Crude protein content of NILs of the four genotypes April Bearded, Bersee, Maris (M.) Huntsman and Maris (M.) Widgeon with different *Rht* alleles. *Rht1* (semi-dwarf), *Rht2* (semi-dwarf), *Rht3* (extreme dwarf), *Rht1+2* (dwarf) and *Rht2+3* (extreme-dwarf) are compared to wild-type *rht* (tall). A, 2021; B, 2022; C, 2023. Each harvest year with three biological replicates (n=3). D, mean of three years and three biological replicates per year (2021, 2022 and 2023; n=9). Significant differences to the control are marked with asterisks (t-test, * $p \leq 0.05$; ** $p<0.01$; *** $p<0.001$). Note: D is the same as Figure 3.

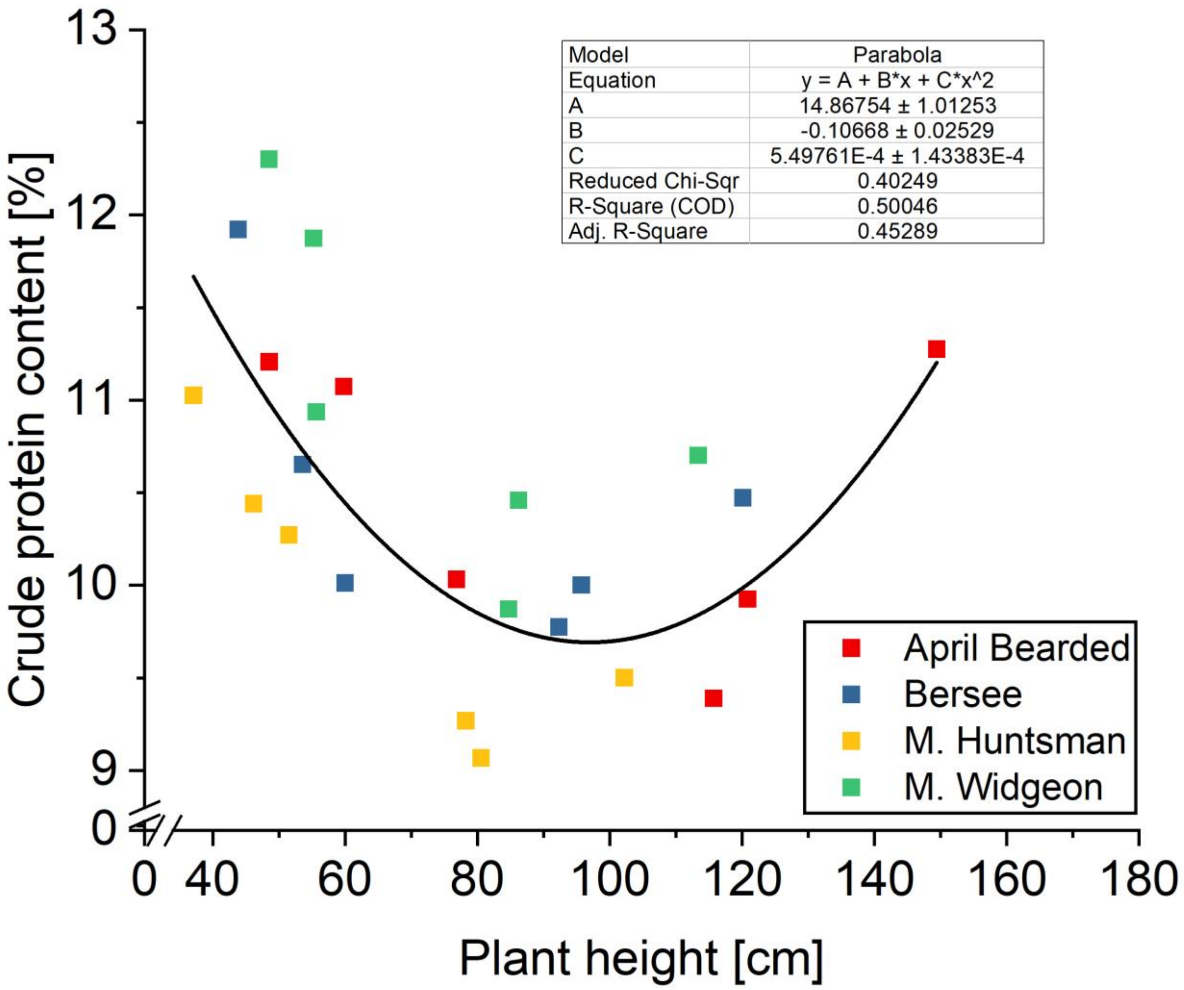

Figure S4: Parabolic fit between crude protein content and plant height of NILs (April Bearded, Bersee, Maris (M.) Huntsman and Maris (M.) Widgeon) with different *Rht* alleles. Mean of three years and three biological replicates per year (2021, 2022 and 2023; *n*=9).

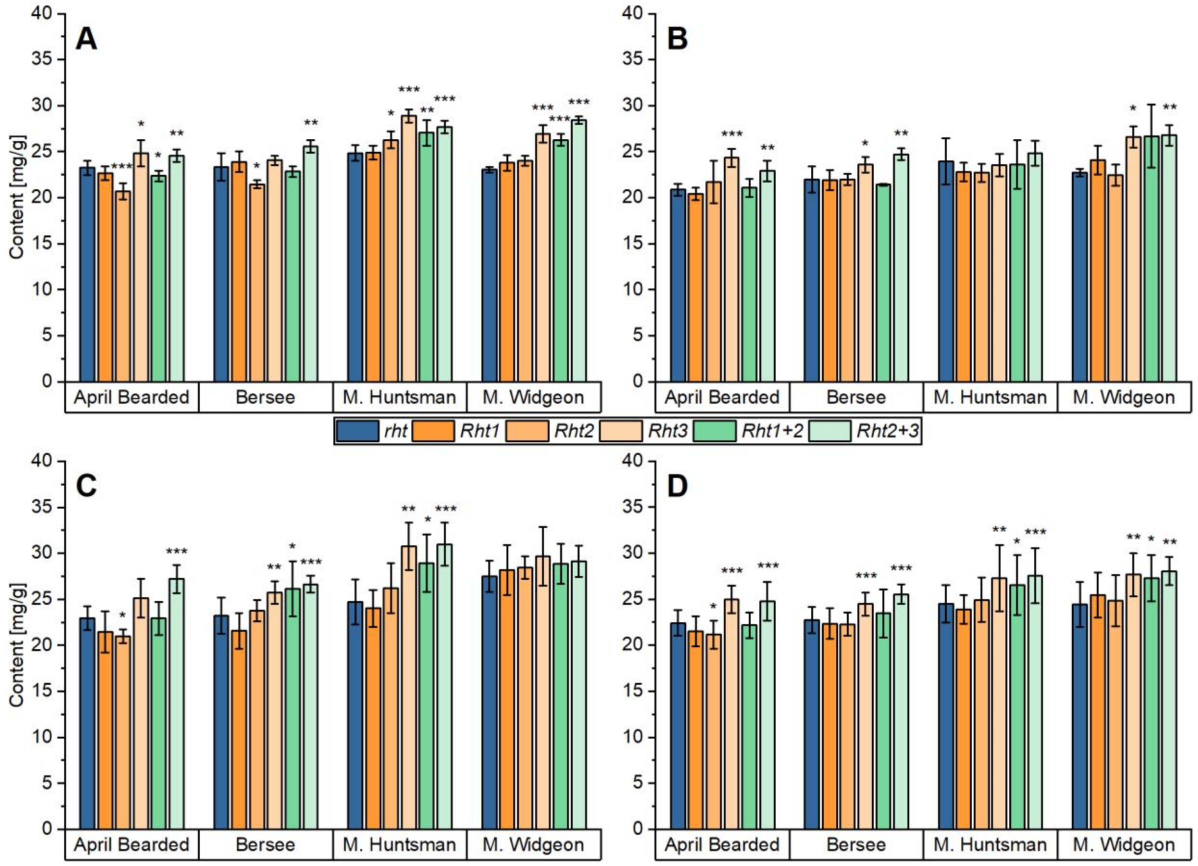

Figure S5: Albumin/globulin content of NILs of the four genotypes April Bearded, Bersee, Maris (M.) Huntsman and Maris (M.) Widgeon with different *Rht* alleles. *Rht1* (semi-dwarf), *Rht2* (semi-dwarf), *Rht3* (extreme dwarf), *Rht1+2* (dwarf) and *Rht2+3* (extreme-dwarf) are compared to wild-type *rht* (tall). A, 2021; B, 2022; C, 2023. Each harvest year with three biological replicates (n=3). D, mean of three years and three biological replicates per year (2021, 2022 and 2023; n=9). Significant differences to the control are marked with asterisks (t-test, * $p\leq0.05$; ** $p<0.01$; *** $p<0.001$).

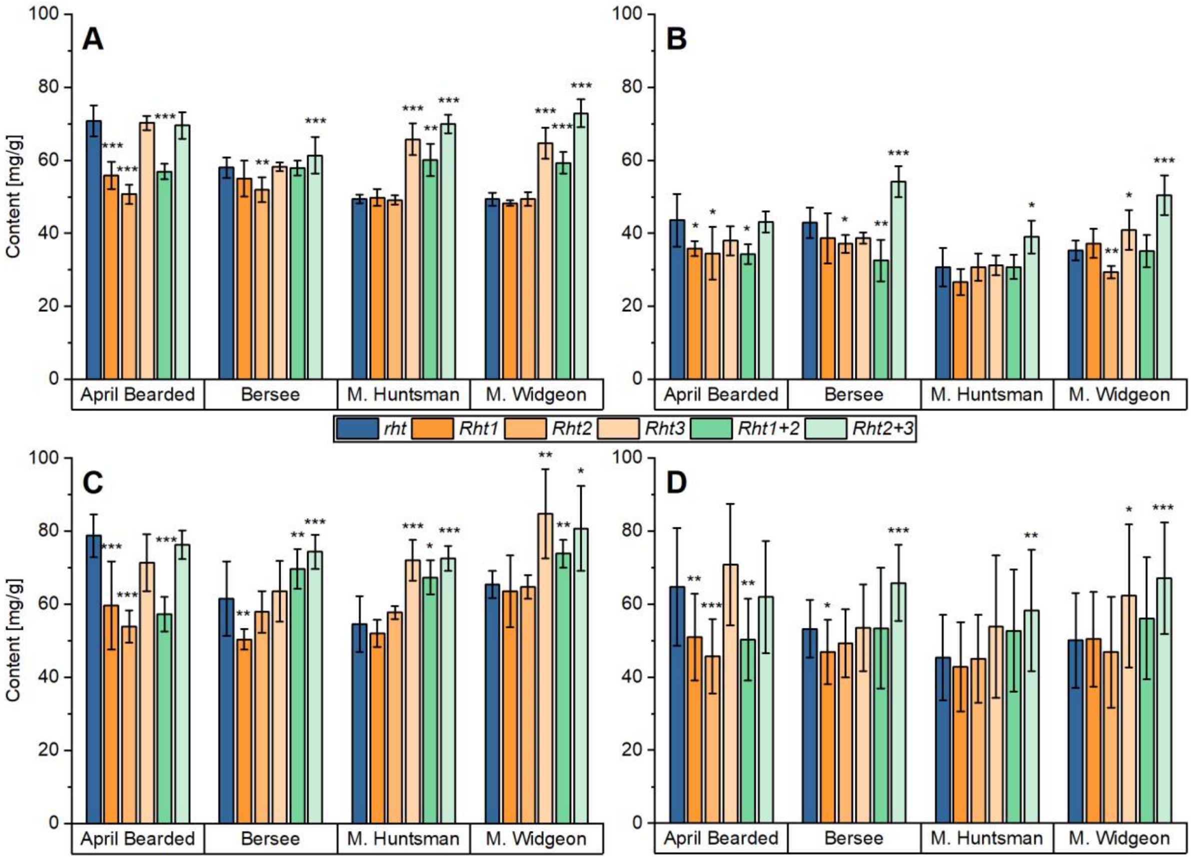

Figure S6: Gliadin content of NILs of the four genotypes April Bearded, Bersee, Maris (M.) Huntsman and Maris (M.) Widgeon with different *Rht* alleles. *Rht1* (semi-dwarf), *Rht2* (semi-dwarf), *Rht3* (extreme dwarf), *Rht1+2* (dwarf) and *Rht2+3* (extreme-dwarf) are compared to wild-type *rht* (tall). A, 2021; B, 2022; C, 2023. Each harvest year with three biological replicates (n=3). D, mean of three years and three biological replicates per year (2021, 2022 and 2023; n=9). Significant differences to the control are marked with asterisks (t-test, * $p \leq 0.05$; ** $p<0.01$; *** $p<0.001$).

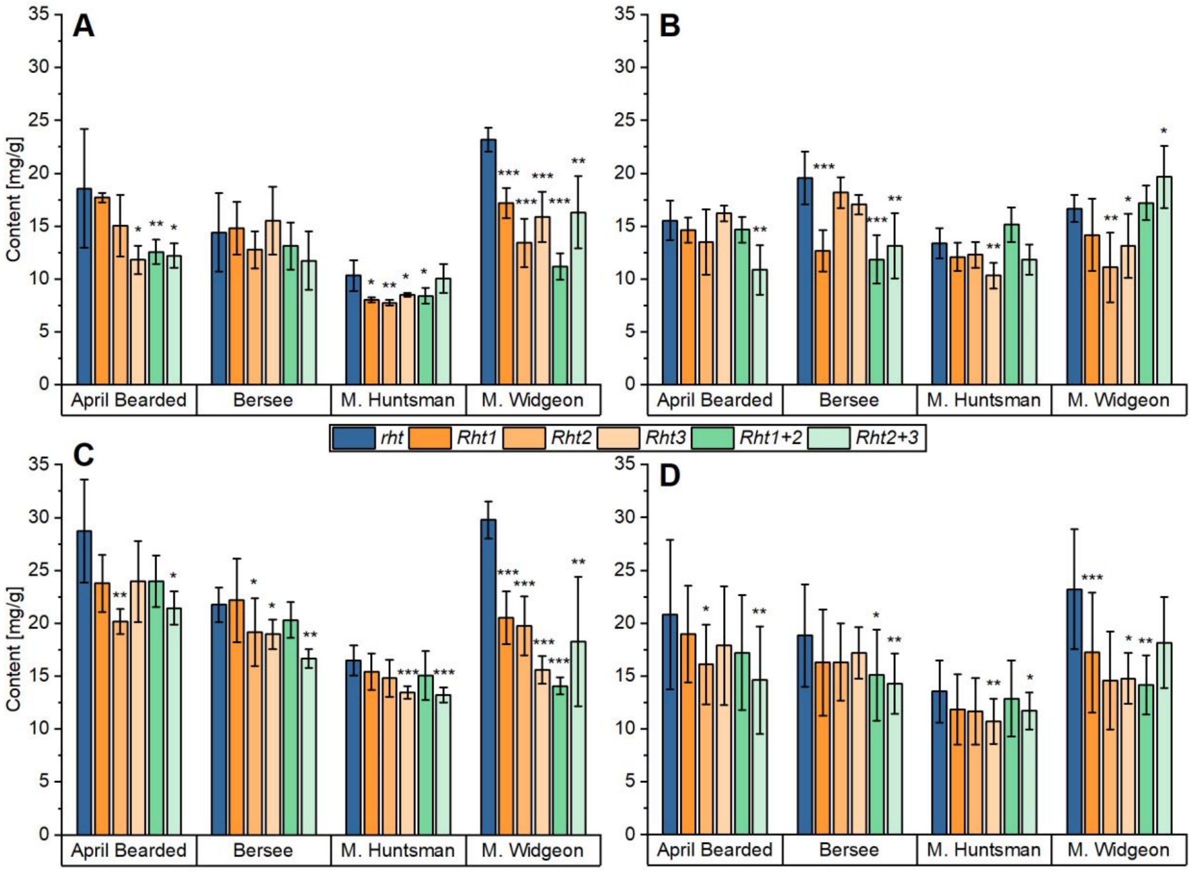

Figure S7: Glutenin content of NILs of the four genotypes April Bearded, Bersee, Maris (M.) Huntsman and Maris (M.) Widgeon with different *Rht* alleles. *Rht1* (semi-dwarf), *Rht2* (semi-dwarf), *Rht3* (extreme dwarf), *Rht1+2* (dwarf) and *Rht2+3* (extreme-dwarf) are compared to wild-type *rht* (tall). A, 2021; B, 2022; C, 2023. Each harvest year with three biological replicates (n=3). D, mean of three years and three biological replicates per year (2021, 2022 and 2023; n=9). Significant differences to the control are marked with asterisks (t-test, * $p \leq 0.05$; ** $p<0.01$; *** $p<0.001$).

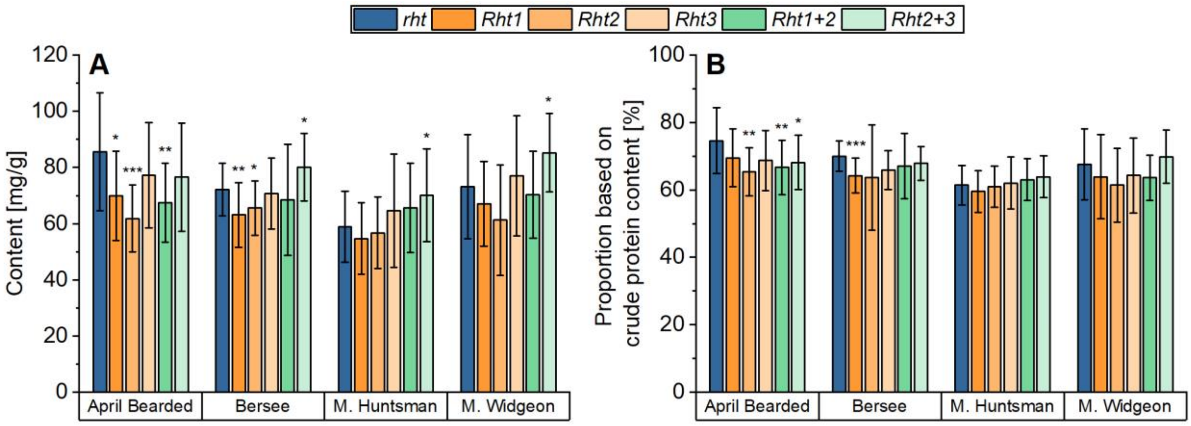

Figure S8: Gluten content (as sum of gliadins and glutenin) of NILs of the four genotypes April Bearded, Bersee, Maris (M.) Huntsman and Maris (M.) Widgeon with different *Rht* alleles. *Rht1* (semi-dwarf), *Rht2* (semi-dwarf), *Rht3* (extreme dwarf), *Rht1+2* (dwarf) and *Rht2+3* (extreme-dwarf) are compared to wild-type *rht* (tall). A, mean of three years and three biological replicates per year (2021, 2022 and 2023; n=9); B, proportion of gluten based on crude protein content. Significant differences to the control are marked with asterisks (t-test, * $p \leq 0.05$; ** $p < 0.01$; *** $p < 0.001$).

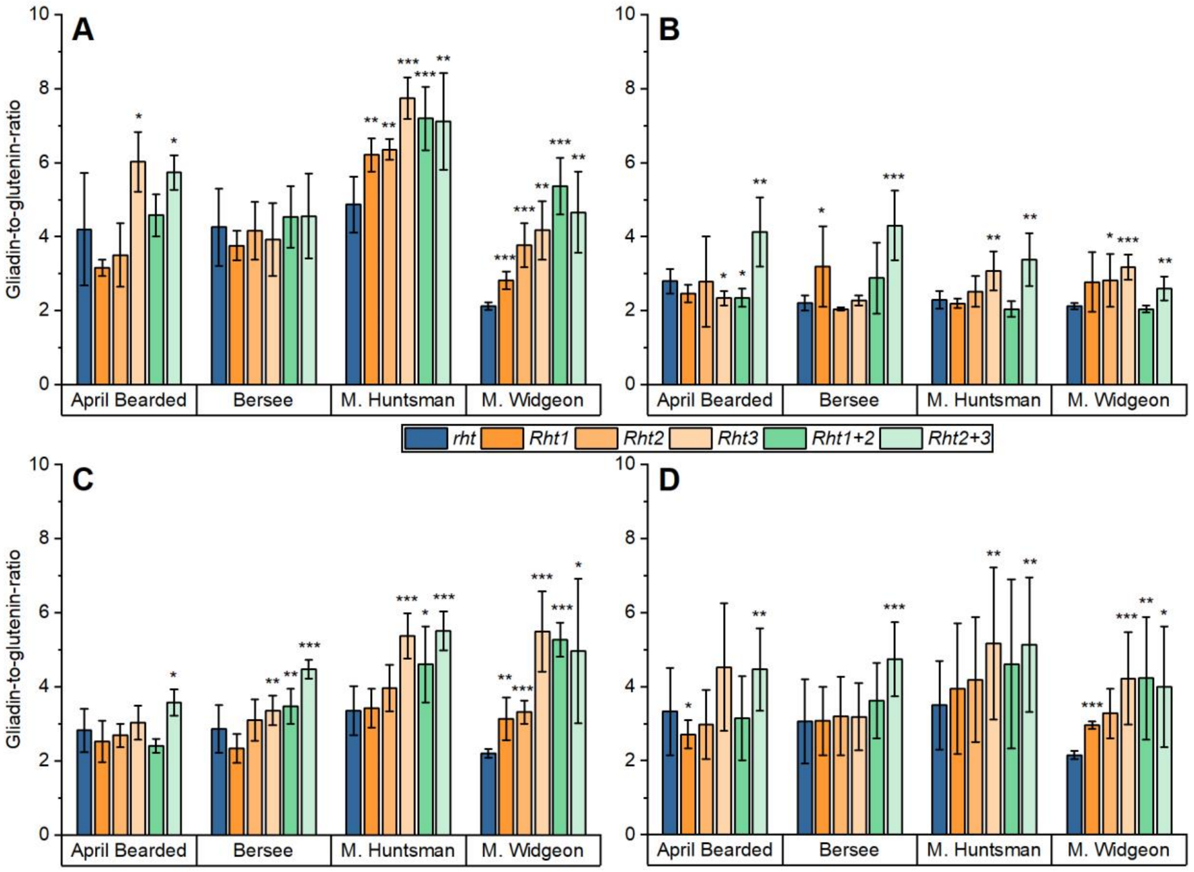

Figure S9: Gliadin-to-glutenin-ratio of NILs of the four genotypes April Bearded, Bersee, Maris (M.) Huntsman and Maris (M.) Widgeon with different *Rht* alleles. *Rht1* (semi-dwarf), *Rht2* (semi-dwarf), *Rht3* (extreme dwarf), *Rht1+2* (dwarf) and *Rht2+3* (extreme-dwarf) are compared to wild-type *rht* (tall). A, 2021; B, 2022; C, 2023. Each harvest year with three biological replicates (n=3). D, mean of three years and three biological replicates per year (2021, 2022 and 2023; n=9). Significant differences to the control are marked with asterisks (t-test, * $p \leq 0.05$; ** $p < 0.01$; *** $p < 0.001$). Note: D is the same as Figure 4D.

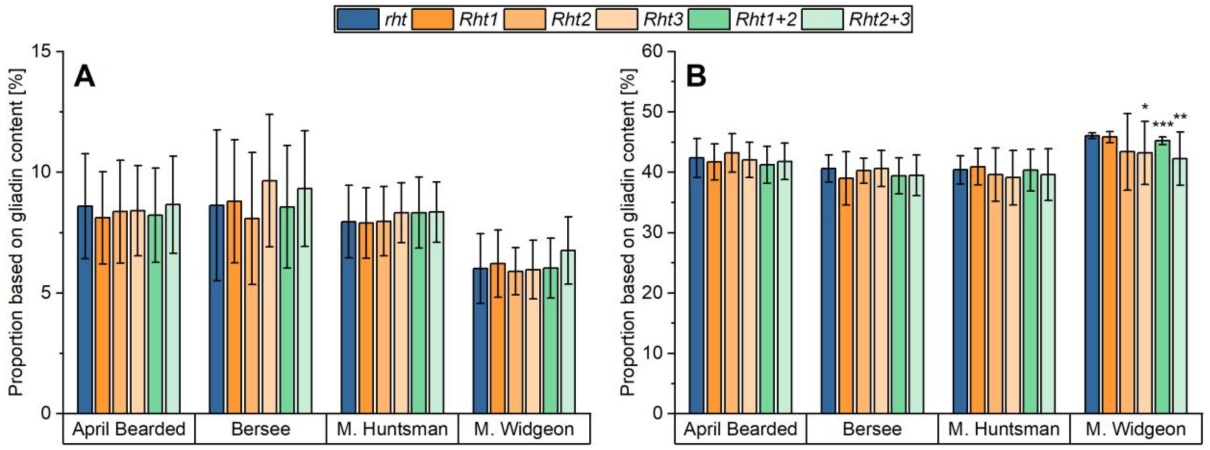

Figure S10: Proportion of gliadin types based on gliadin content of NILs of the four genotypes April Bearded, Bersee, Maris (M.) Huntsman and Maris (M.) Widgeon with different *Rht* alleles. *Rht1* (semi-dwarf), *Rht2* (semi-dwarf), *Rht3* (extreme dwarf), *Rht1+2* (dwarf) and *Rht2+3* (extreme-dwarf) are compared to wild-type *rht* (tall). A, ω1,2-gliadins; B, γ-gliadins. Mean of three years and three biological replicates per year (2021, 2022 and 2023; n=9). Significant differences to the control are marked with asterisks (t-test, * $p \leq 0.05$; ** $p < 0.01$; *** $p < 0.001$).
